# HAVOC: Small-scale histomic mapping of cancer biodiversity across large tissue distances using deep neural networks

**DOI:** 10.1126/sciadv.adg1894

**Published:** 2023-09-29

**Authors:** Anglin Dent, Kevin Faust, K. H. Brian Lam, Narges Alhangari, Alberto J. Leon, Queenie Tsang, Zaid Saeed Kamil, Andrew Gao, Prodipto Pal, Stephanie Lheureux, Amit Oza, Phedias Diamandis

**Affiliations:** ^1^ Department of Laboratory Medicine and Pathobiology, University of Toronto, Toronto, ON M5S 1A8, Canada.; ^2^ Department of Computer Science, University of Toronto, 40 St. George Street, Toronto, ON M5S 2E4, Canada.; ^3^ Princess Margaret Cancer Centre, 101 College Street, Toronto, ON M5G 1L7, Canada.; ^4^ Department of Pathology and Laboratory Medicine, David Geffen School of Medicine, University of California Los Angeles, Los Angeles, CA, USA.; ^5^ Laboratory Medicine Program, Department of Pathology, University Health Network, 200 Elizabeth Street, Toronto, ON M5G 2C4, Canada.; ^6^ Department of Medical Biophysics, University of Toronto, 101 College St, Toronto, ON M5G 1L7, Canada.

## Abstract

Intratumoral heterogeneity can wreak havoc on current precision medicine strategies because of challenges in sufficient sampling of geographically separated areas of biodiversity distributed across centimeter-scale tumor distances. To address this gap, we developed a deep learning pipeline that leverages histomorphologic fingerprints of tissue to create “Histomic Atlases of Variation Of Cancers” (HAVOC). Using a number of objective molecular readouts, we demonstrate that HAVOC can define regional cancer boundaries with distinct biology. Using larger tumor specimens, we show that HAVOC can map biodiversity even across multiple tissue sections. By guiding profiling of 19 partitions across six high-grade gliomas, HAVOC revealed that distinct differentiation states can often coexist and be regionally distributed within these tumors. Last, to highlight generalizability, we benchmark HAVOC on additional tumor types. Together, we establish HAVOC as a versatile tool to generate small-scale maps of tissue heterogeneity and guide regional deployment of molecular resources to relevant biodiverse niches.

## Teaser

Deep learning-based tissue morphometric analysis identifies regional intra-tumoral heterogeneity to guide molecular analysis.

## INTRODUCTION

Tumoral heterogeneity underpins modern frameworks of tumor evolution and treatment resistance, but resolving cancer biodiversity distributed across long distances in resection specimens has proven challenging (*1*–*3*). Even small biopsies (~1 cm^3^) can contain ~10^8^ tumor cells, a number representing many orders of magnitude more than what current single-cell profiling approaches can routinely process (~10^3^ cells) (*4*–*6*). Similarly, asymmetric distributions of biological variation across larger specimens can result in nonrepresentative undersampling and/or mixing of critical tumor subpopulations using bulk-based expression profiling approaches (*7*, *8*). These complexities may be further amplified in large cohort studies and analysis of recurrent tumor specimens where variations in cellular composition across samples can complicate interpretations (*9*–*11*).

**Fig. 1.**
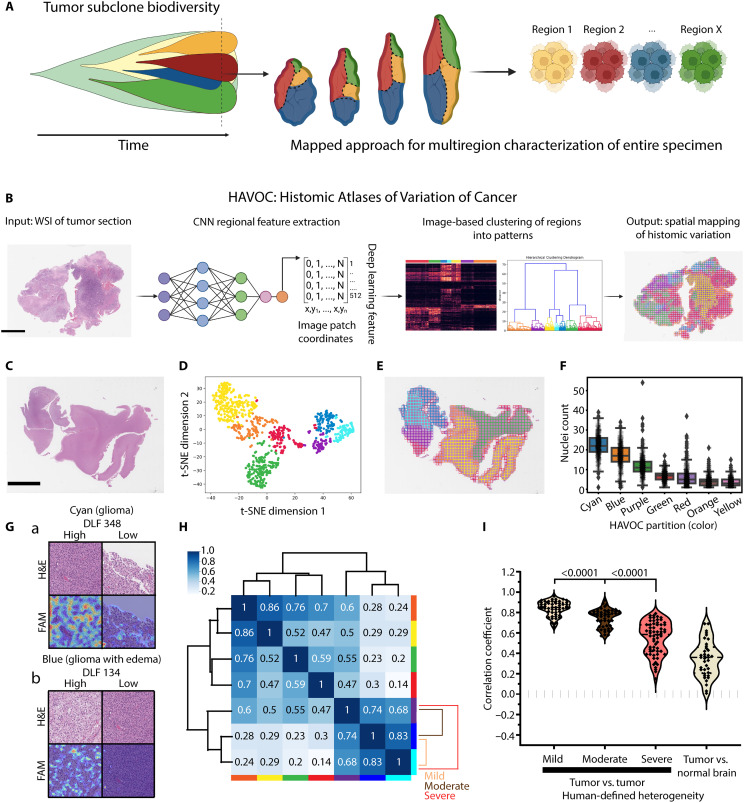
Mapping biodiversity in cancer tissue with HAVOC. (**A**) Cartoon depicting regional evolution of intratumoral subclones and a map-guided approach to sampling. (**B**) HAVOC workflow summary. A pretrained CNN is used as a morphology feature extractor for input images. The generated DLFVs of individual image patches (tiles) are then used to carry out image feature-based clustering and map spatial coordinates to distinct histomic fingerprints back to the original WSI. (**C** to **E**) Representative mapping of a diffuse glioma. The relative spectrum of morphologic patterns of image tiles can be explored by dimensionality reduction (e.g., t-SNE) and clustering. Both highlight distinct tumor (blue, purple, and cyan) and nontumoral (yellow, green, and red) regions. Scale bar, 6 mm. (**F**) Strip boxplot of Mask R-CNN–based quantifications of nuclei per HAVOC region. The boxplot shows minimum, first quartile, median, third quartile, and maximum along with outliers (diamonds). Counts represent nuclear instances per 4218 μm^2^. (**G**) Mapping of individual DLFs overrepresented in these HAVOC-defined tumor regions highlights interpretable morphological patterns that correlate with these defined glioma niches (e.g., tumor nuclei and edema, respectively). Dimensions of image patches shown: 0.27 mm^2^. (**H**) Pairwise Pearson correlation coefficients (*r*) of the DLFVs of HAVOC-defined partitions in (E), highlighting inverse correlation with the degree of morphological heterogeneity across this representative case. Sample pairs of mild/moderate/severe correlations are provided. (**I**) Violin plots showing concordance between HAVOC *r* values and semiquantitative assessments of regional heterogeneity (e.g., mild, moderate, or severe). DLFV *r* of lesional versus nonlesional regions included as reference.

In geography, large-scale maps that provide high-resolution information of relatively small topographic areas (e.g., cities and provinces) are often complemented with smaller-scale cartographic surveys that document specific sets of relevant features over larger regions (e.g., countries and continents) (*12*, *13*). In such disciplines, these small-scale atlases aid in guiding systems-level decisions and resource allocation (e.g., nature conservation efforts). In the context of cancer, systematic approaches to establishing coordinates of potential biodiversity across large tissue specimens could guide deployment of limited molecular profiling resources to better capture tumor-level heterogeneity (Fig. 1A) (*14*).

To address this critical challenge, we developed HAVOC, a histology-based deep neural network pipeline aimed at creating “Histomic Atlases of Variation Of Cancers” and providing spatially contextualized estimates of biodiversity across virtually any scale (Fig. 1B). This approach leverages both classic and modern dogmas of histopathology (i) that substantial changes in molecular programs are often accompanied with modifications in cellular morphologies and (ii) that such cytoarchitectural variations can be objectively defined by contemporary computer vision strategies (*15*–*18*). Previous studies exploring intratumoral heterogeneity using computer vision have largely used supervised approaches, in which neural networks are trained using hematoxylin and eosin (H&E)–stained whole slide images (WSIs) with genetically defined ground truth labels generated from bulk tumor tissue [e.g., The Cancer Genome Atlas (TCGA)] (*19*–*21*). Once trained, these models are then applied to predict the mutational, cell composition, and transcriptional status of individual image patches from larger tissue areas. While such approaches have shown capacity to predict the presence of immune infiltrates and a handful of specific mutations in a context-specific manner, more intricate tumor cell–intrinsic biological programs (e.g., proliferation, hypoxia, and DNA repair) have proven more challenging to generalize across most tumor types (*22*–*24*). Similarly, such approaches usually require knowledge and design around specific mutations defined a priori (e.g., FGFR3 mutations in bladder cancer) and may therefore be less suitable for screening for potential intratumoral heterogeneous subclones that may be patient-specific and emerge downstream of these common initiating genetic events (*25*, *26*). To address this, one recent study used deep learning to detect immune and stroma infiltrates and leveraged these features as geographic guideposts to define immune “cold” and “hot” regions of non–small cell lung carcinomas. Using whole-exome and RNA sequencing (RNA-seq) on these deep learning–defined regions, the authors showed that cancer subclones derived from immune cold regions shared closer proximity in mutational space than subclones from immune hot regions (*14*). Such molecularly agnostic and prospective mapping approaches of geospatial variability are critical as they may provide more personalized and precise insights into the emergence of treatment-resistant subclones and aggressive clinical phenotypes (*27*, *28*).

Here, we show that by using unsupervised partitioning of patterns found on H&E-stained WSIs, HAVOC can provide coordinates and relative estimates of relevant morphologic and molecular patterns of cancer variation. This approach does not require any a priori framework of biodiversity, allowing for tumor heterogeneity to be explored free of any predefined constraints (e.g., regional lymphocytic infiltrates) or narrow molecular definitions (e.g., specific mutations and aneuploidy) that may not robustly generalize in molecularly heterogeneous cancers. Instead, we use the working assumption that while morphological patterns of complex tumor tissue may not be entirely specific of underlying genetic features or biology across patients, relative intratumoral differences in cytoarchitectural profiles are often driven by or result in meaningful molecular differences. This phenomenon can be leveraged as an entry point for spatial stratification of regions of interest by HAVOC, followed by the dedicated molecular characterization of these coordinates of variation. In practical terms, the more morphologically distinct regions are, the higher the chances that they harbor divergent functional differences.

Specifically, using high-grade gliomas as a proof of concept, an aggressive form of brain cancer that can show substantial intratumoral variation, HAVOC’s predictions of biovariation showed strong concordance with both human-defined and molecularly defined estimates of heterogeneity. This regional analysis also specifically revealed that tumor subpopulations with varying degrees of phenotypic differentiation and proliferative capacity can often coexist and be geographically separated in high-grade gliomas, underscoring the need for more intelligent sampling strategies. The unique spatially defined glioma proteomic dataset generated in this study also showed additional patient-specific regional differences that can be further explored in real time in the Brain Protein Atlas (https://www.brainproteinatlas.org/dash/apps/ad) (*29*). We further highlight the generalizability of HAVOC to other tumor types and cancer models and show that it can be deployed without the need for any additional context-specific training. In addition to the available code for local implementation, we developed a server version of HAVOC that can be accessed via a web interface (https://www.codido.co) without the need for any specialized hardware or software expertise. Routine generation of such small-scale histomic atlases of biologic variations in cancer offers an opportunity to better identify and document critically divergent cellular habitats across large geographic regions and may aid in better managing tumor heterogeneity for both large cohort studies and personalized medicine efforts.

## RESULTS

### Prediction and relative quantification of histomorphological heterogeneity by HAVOC

To assess whether delineation and quantification of spatial transitions in histomorphology could be automated, we applied an unsupervised image clustering framework (*30*) to a digitized WSI cohort of brain tumors comprising largely of diffuse gliomas (*n* = 40), showing varying degrees of regional heterogeneity. The advantage of this unsupervised deep learning strategy is that it helps overcome the substantial case-to-case morphologic variability that is seen in many malignant cancers such as high-grade gliomas (*31*). Briefly, WSIs are individually partitioned into 0.066- to 0.27-mm^2^ nonoverlapping image patches that approximate the field of view diameter of a 40× to 100× objective on a standard modern microscope for analysis. We found that this magnification offers a consistent and favorable balance of both individual cellular differences (e.g., nuclear features and cellularity) and more complex secondary tumor structures (e.g., infiltration and glands/rosette formation) that help define specific tumor types and biology. We note, however, that these patch dimensions are completely tunable based on the context and applications (see Materials and Methods for more discussion). These generated image patches are then passed through and analyzed by a histology-optimized convolutional neural network (CNN) previously trained on a diverse set of nearly 1 million pathologist-annotated image patches extracted from more than 1000 brain tumors (*15*, *32*). Rather than carrying out classification, the 512-dimensional deep learning feature vector (DLFV), generated in the penultimate layer of the CNN, is extracted and used as a “histomic” feature set. These DLFV signatures are then used to carry out patch-level clustering (*k* = 2 to 9) and spatially map morphologic variation across entire WSIs (Fig. 1, C to E, and fig. S1). In addition to lesion segmentation from normal brain tissue elements in early partitions, when present, this approach also nonrandomly segregated tumor areas with progressively more subtle regional morphologic pattern differences in later subdivisions {e.g., variations in tumor cellularity and intratumoral edema, *P* ≤ 1.2 × 10^−11^; cellularity [Mask R-CNN–based nuclear quantification, analysis of variance (ANOVA)] and positional coordinates of partitioned tiles (Kolmogorov-Smirnov)} (Fig. 1F and fig. S2). Generation of feature activation maps (FAMs) of salient individual deep learning features (DLFs), enriched in each compartment, provided further qualitative support and insight of human-perceivable histomorphologic patterns (e.g., cellularity and edema) that were associated and/or contributing to each generated HAVOC partition (Fig. 1G). Pearson correlation coefficients (*r*) of regional DLFVs (averaged over tiles within a HAVOC partition) correlated with categorical human estimates of histologic variation (overall assessment between paired HAVOC regions), allowing this metric to serve as a quantitative approximation of the degree of variability across HAVOC-defined regions (Fig. 1, H and I; *n* = 40 cases with 200 total comparisons; *P* < 0.0001, Mann-Whitney *U* test). As expected, comparison of normal tissue and tumor regions had the lowest DLFV *r* (DLFV *r*_mean_ = 0.35; *n* = 37 comparisons). This DLFV *r* score also showed progressively increasing values between pairs of tumor regions in which their relative heterogeneity was scored as “severe” [morphologic differences observable at low power (5× to 10×), DLFV *r* = 0.55; *n* = 62], “moderate” [morphologic differences appreciable at only high magnification (20× to 40×), DLFV *r* = 0.75; *n* = 49], and “mild” (subtle/subjective morphological differences between regions even at high magnification, DLFV *r* = 0.84; *n* = 52). While we found that the optimal number of subdivisions varied depending on the degree of tissue complexity present on individual slides, for most cases, the majority of human-discernible patterns of histomorphologic variation (defined by the “moderate-severe” categories and corresponding DLFV *r* < 0.74) plateaued and reached saturation after seven to eight partitions, irrespective of the overall level of heterogeneity found on the WSI (fig. S3). Together, these data highlight how HAVOC can provide an automated, objective, and human-concordant tool to estimate spatial histomorphologic variation across cancer tissue.

### Spatial morphologic fingerprints align with molecular patterns of heterogeneity

**Fig. 2.**
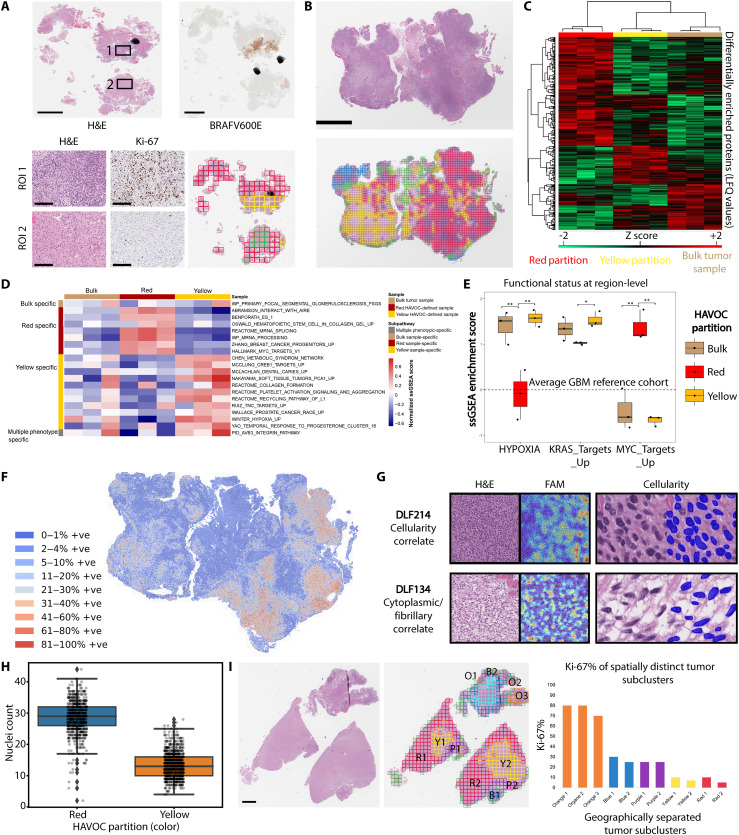
HAVOC-defined partitions align with regional biodiversity. (**A**) Histopathology images of an IDH–wild-type glioblastoma demonstrating a BRAFV600E-mutated hyperproliferative subclone resolved by immunohistochemistry. This molecularly distinct subpopulation was also resolved by HAVOC (yellow partition). Scale bar, 2 mm. (**B** to **E**) LC-MS/MS profiling of HAVOC-defined partitions and bulk tissue samples shows distinct proteomic profiles. (B) HAVOC delineated four distinct regions in this glioblastoma section. The two large cellular tumor regions (red/yellow) were excised and analyzed by LC-MS/MS. Scale bars, 4 mm. (C) Hierarchical clustering of the resolved regional proteomes highlights differences in protein expression of HAVOC partitions and bulk samples (*n* = 3 replicate tissue sections). (D) Normalized ssGSEA scores of 64 GBM-informative signatures (*45*) across the different HAVOC regions and bulk tissue. (E) Boxplots more specifically highlighting regional differences in the functional status of three key protein-level programs in glioblastoma [MYC_targets (proliferation), KRAS_targets (invasion), and Hypoxia (*33*)]. Bulk signatures provided for reference. Enrichment scores were inferred with existing XGBoost models, and the dotted line represents average values from the training GBM cohort (*33*). (**F**) Spatial proliferation index differences (as assessed via CNN-based quantification of Ki-67) spatially align with the different HAVOC partitions. (**G**) Feature activation mapping defined morphologic correlates of the profiled HAVOC partitions. Partitions showing proliferative and invasive biology show high cellularity and cytoplasmic/fibrillary patterns, respectively. Dimensions of image patches shown: 0.27 mm^2^. Sample masks from the Mask R-CNN cellularity analysis provided for reference (dimension: 4218 μm^2^). (**H**) Strip boxplot of Mask R-CNN–based quantifications of nuclei/HAVOC region. The boxplot shows minimum, first quartile, median, third quartile, and maximum along with outliers (diamonds). Counts represent nuclear instances per 4218 μm^2^. (**I**) Representative H&E and HAVOC map of an IDH–wild-type glioblastoma section, with geographically separated subclusters (e.g., O1, O2, and O3) showing similarly grouped histomic (HAVOC) signatures. Scale bars, 2 mm.

In one of the glioblastoma cases of our initial cohort, we noted that HAVOC partitioning captured a BRAFV600E-mutated tumor subclone, showing an elevated Ki-67 (MIB1) proliferation index on immunohistochemistry (Fig. 2A). This supported the possibility that regional changes in morphologic fingerprints may also predict relevant phenotype-level variability in tumor biology. To begin formally testing whether histomic heterogeneity, perceived by HAVOC, also correlated with global molecular differences, we compared DLFV *r* values across the major histomorphologic hallmarks of diffuse gliomas including regions of high cellularity (CT), infiltrating tumor (IT), and brain tissue at the leading tumor edge (LE) to proteomic and transcriptional profiles generated from these areas in previous studies (fig. S4) (*33*, *34*). HAVOC-defined niche variations correlate with proteomic (*r*^2^ = 0.79) and transcriptomic (*r*^2^ = 0.89) profiles, with similar glioma histomorphologic regions (e.g., multiple IT regions) having a higher degree of DLFV and molecular similarity when compared to more distinct niches (e.g., CT versus LE regions).

To more directly assess the predictive power of HAVOC for resolving distinct spatial expression profiles, we next determined whether regional histomically defined niches, exclusively within the cellular glioma compartment, could predict protein-level heterogeneity. We focused our regional analysis on proteomic outputs due to known proteogenomic discordances in many cancers in which genetic changes may not always align with downstream cellular protein–driven phenotypes (*35*–*37*). Laser capture microdissection (LCM) followed by liquid chromatography–tandem mass spectrometry (LC-MS/MS) analysis of cellular tumor regions highlighted substantial regional variability and numerous differentially encoded proteins (false discovery rate = 0.1, *n* = 3 separate microdissection replicates) (Fig. 2, B and C; figs. S5 and S6; and tables S1 and S2). Pathway analysis, using a previously defined set of 64 proteogenomically concordance signatures (*33*), highlighted regional heterogeneity (RED versus YELLOW neighboring regions) in proliferative (MYC-, *P* = 0.0006; Tukey method), invasive (KRAS-, *P* = 0.03; Tukey method), and hypoxic (*P* = 0.0048; Tukey method) programs (*33*), among others (Fig. 2, D and E). Notably, the marked regional variability of some of these critical programs in the RED partition was completely lost when the entire tissue section was profiled (“bulk”), underscoring the value of multiregion sampling of highly heterogeneous tumors when using expression-based techniques (MYC- Red versus Bulk, *P* = 0.001; Hypoxia- Red versus Bulk, *P* = 0.008; Tukey method) (Fig. 2E). The two major HAVOC partitions from patient IV of the main study cohort showed a narrower profile of differences in functional programs (e.g., hypoxia), highlighting the capacity of HAVOC to detect even subtle molecular differences across individual cases driven by the microenvironment (*P* = 0.04, *t* test) (fig. S6B). Overall, this highlights how predicted biological programs can markedly change in adjacent regions and may not be well captured by profiling techniques that either focus on the overall (bulk) or very confined tissue regions.

Given the role of MYC in cell cycle progression, we validated these differences in the case presented in Fig. 2B using CNN-based estimates of Ki-67 staining. This confirmed marked spatial variation in the proliferative capacity of these adjacent regions (Fig. 2F; *P* < 0.0001, *t* test). We further explored the predictive potential of HAVOC for heterogeneously proliferating subclones in another independent cohort of glioblastomas assembled to contain cases that displayed regional variations in Ki-67 (*n* = 5, table S2). In all these cases, HAVOC’s heterogeneity maps defined H&E-based DLFV signatures that correlated with partitions showing differential intratumoral rates of proliferation (*P* < 0.0001, *t* test; fig. S7). In addition to the relationship where regions with higher DLFV *r* showed more comparable proliferation rates, comparison to the overall proliferation index across the entire WSI also showed important differences. This was especially true in failing to capture information regarding the most aggressive and highly proliferative tumor foci within these heterogeneous tumor samples. Given that they represent a relatively small fraction of the overall tumor specimen, these highly proliferative regions are likely also to be missed with even random multiregion sampling. These results further underscore how morphologic profiles can guide attention to even small biologically distinct regions that may play an important role in tumor evolution and treatment resistance.

To more directly explore some of the potential correlations between proliferation programs and morphology, we next assessed whether specific DLFs were being activated by discernible histomorphologic features within regions displaying distinct Ki-67 indices in a representative case (fig. S8). FAMs of regionally enriched DLFs highlighted a transition in pattern with areas with high cellularity (DLF214; activation on tumor nuclei) to regions displaying fibrillar cytoarchitecture (DLF134; activation on cytoplasmic processes) in variably high to low Ki-67–positive regions, respectively (Fig. 2G). We validated this qualitative observation again using a Mask R-CNN nuclear instance segmentation model to quantify cellular differences between these HAVOC partitions (*t* = 69.6; *P* = 0, *t* test) (Fig. 2, G and H).

We also noted that in some cases of the validation cohort, HAVOC partitions [e.g., Orange (O)] often formed isolated “subclusters” (e.g., O1, O2, and O3) that while spatially separated showed similar morphologic patterns (e.g., hypercellularity and infiltrative phenotype) and maintained stable cluster-specific Ki-67 proliferation indices (Fig. 2I). These findings, that geographically distinct tissue regions can be grouped together, suggested that H&E-based DLFV signatures may generalize to multiple spatially separated tumor subregions and could help construct maps of biovariation even across larger specimens spanning multiple histopathological slides.

### Mapping biovariation across centimeter-scale distances and larger specimens with HAVOC

**Fig. 3.**
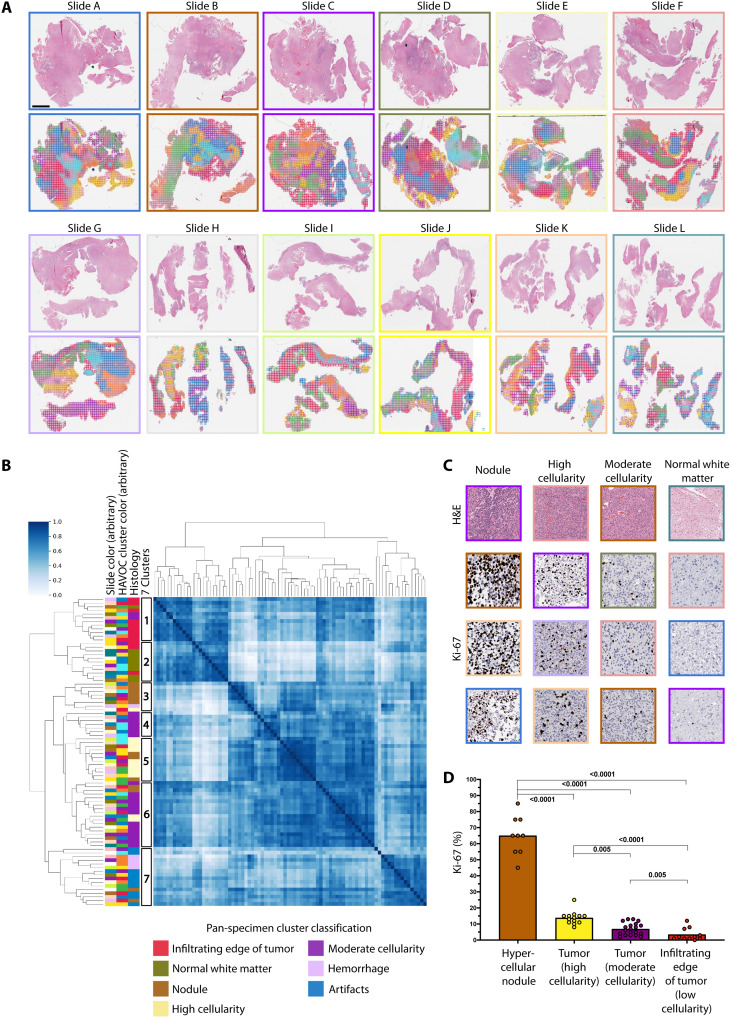
Small-scale mapping of biovariation across an entire tumor specimen using HAVOC. (**A**) Twelve sequential H&E sections generated from a recurrent IDH-mutated, 1p19q- codeleted oligodendroglioma measuring 5.4 cm × 4.1 cm × 1.8 cm in dimensions with accompanying HAVOC heterogeneity maps. Scale bar, 4 mm. The assigned slide color scheme is arbitrary. (**B**) Pairwise Pearson correlation matrix arranging all 84 clusters shown in (A) in a hierarchical fashion. Slide colors correspond to the same colors shown in the former panel. There is significant distribution enrichment of human-annotated morphologic patterns within different subclusters defined by the hierarchical tree. A seven-cluster solution is shown with additional solutions offered in fig. S10. There was no significant enrichment of a specific slide in any of the proposed clusters (chi-square test; please see table S3 for contingency tables and associated *P* values) (**C**) Representative images of Ki-67 (MIB1) from the different clustered regions. Image patch border colors correspond to their originating WSI Slide color assigned in (A). Dimensions of image patches shown: 0.27 mm^2^. (**D**) Histogram of estimated Ki-67 (MIB1) across different histologically defined regions (*P* values generated using Mann-Whitney *U* test). Note: Color of histogram bars aligns with the color legend shown in (B).

Given the ability of histomic signatures to group geographically separated tissue regions with similar biology on individual slides (Fig. 2I), we next assessed the generalizability of this concept to the organization of tumor heterogeneity within individual large tissue specimens spanning multiple slides. As an example, we highlight a HAVOC map generated on a large recurrent isocitrate dehydrogenase (IDH)–mutated 1p19q-codeleted anaplastic oligodendroglioma, central nervous system (CNS) World Health Organization (WHO) grade 3, showing heterogeneous radiographic signal and measuring 4.4 cm in maximum dimensions. To map the histomorphologic heterogeneity across this entire case, we first generated seven separate HAVOC partitions for each of the 12 H&E sections spanning the entire resected specimen (5.4 cm × 4.1 cm × 1.8 cm ) (Fig. 3A). This was meant to ensure that most of the spatial heterogeneity found on each WSI would appropriately be sampled and independently annotated by our team (see fig. S3). The resulting 84 HAVOC-defined regions then underwent hierarchical clustering to help organize them across the entire specimen based on their pairwise DLFV similarities (*r*) (Fig. 3B). Even across multiple WSIs, regions clustering together appeared to be more concordant with histomorphologic annotations rather than their positional coordinates (“Histology” versus “Slide” color track) (Fig. 3, B and C, and fig. S9).

Overall, by examining the hierarchical clustering dendrogram pattern and their enrichment for specific annotated histomorphologies (chi-square test), we found support for the presence of 2, 3, and 7 relevant pan-sample subgroupings (Fig. 3B, fig. S10A, and table S3). In the broadest sense, we found seven major clusters enriched in brain tissue neighboring the tumor (“infiltrating edge of tumor,” cluster 1; *P* = 1.6 × 10^−6^, χ^2^ test), white matter (cluster 2; *P* = 7.4 × 10^−9^, χ^2^ test), hypercellular tumor regions with nodularity (cluster 3; *P* = 5.1 × 10^−4^, χ^2^ test), tumor with moderate (clusters 4 and 6; *P* = 5.0 × 10^−3^ and *P* = 3.8 × 10^−3^, χ^2^ test) to high cellularity (cluster 5; *P* = 4.4 × 10^−7^, χ^2^ test), and hemorrhage and artifacts (e.g., clusters composed largely of tiles with tissue edges) (cluster 7; *P* = 4.8× 10^−10^, χ^2^ test) (Fig. 3B and table S3). Alternative clustering solutions highlighted the ability to resolve largely uninvolved/intermixed brain tissue (cluster 1; *P* = 6.3 × 10^−10^, χ^2^ test), cellular neoplastic regions (cluster 2; *P* = 2.6 × 10^−5^, χ^2^ test), and noncontributory tissue (cluster 3; *P* = 4.8 × 10^−10^, χ^2^ test) across the entire specimen. Last, within the tumor-enriched cluster, HAVOC displayed a robust ability to separate out regions with hypercellular nodularity (cluster 1; *P* = 8.9 × 10 ^−4^, χ^2^ test) from other less cellular regions (cluster 2; *P* = 3.1 × 10^−1^, χ^2^ test). This final arrangement was further objectively validated with substantially different estimated proliferation indices (Ki-67) between multiple nodular containing and nonnodular cellular tumor regions found in the respective cluster groups (*P* < 0.005, Mann-Whitney *U* test; Fig. 3, C and D). The positional *t*-distributed stochastic neighbor embedding (t-SNE) mapping arrangement of all 10,973 0.27-mm^2^ image patches (*38*) from this lesion also supported this, with a gradient of morphological patterns, transitioning from nodular, hypercellular, moderately cellular, and nontumor/low cellularity tumor regions (fig. S10B). In addition to providing additional support for the ability to automate mapping of histomorphologic patterns across large tissue areas, this final analysis also highlighted alternative promising approaches to carrying out multi-WSI analyses (see Materials and Methods for additional discussion).

**Fig. 4.**
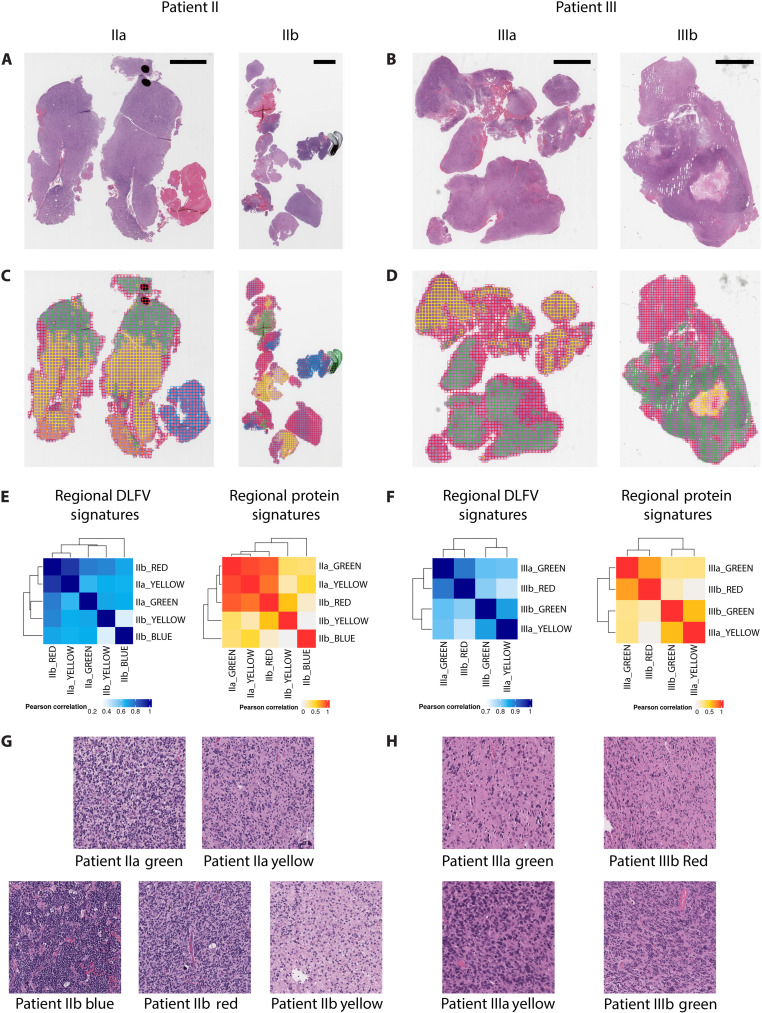
HAVOC mapping across related WSI pairs aligns with overall molecular correlations. (**A** and **B**) Paired H&E WSI sections from two independent individuals with IDH–wild-type high-grade gliomas. (**C** and **D**) Respective HAVOC partitions across respective paired slides. (**E**) Multislide DLFV correlation matrices across both slides of patient II define a distinct focal hyperdense outlier region (sample IIb blue cluster). These multislide map distances are in agreement with global patterns of proteomic variations derived from each of the major HAVOC-defined partitions. (**F**) Another example of HAVOC multislide regional partitions that identified reciprocally aligned regions across slide pairs. These HAVOC-defined interslide niche similarities are also in agreement with the intratumoral proteomic variations of this specimen. (**G** and **H**) Representative image patches from HAVOC partitions provided to highlight that the magnitude of the qualitative morphological differences is in agreement with the relative HAVOC and molecular differences. Dimensions of image patches shown: 0.27 mm^2^.

To further examine whether HAVOC could generalize to identify regional molecular profiles across larger tumor distances, we carried out this multislide mapping and LC-MS/MS profiling workflow on paired slides from three additional IDH–wild-type high-grade gliomas (histology: two WHO grade 4 and one WHO grade 3) (Fig. 4, A and B; fig. S11; and table S1). In one case, HAVOC appropriately defined a focal hyperdense outlier region from other tumor morphologies (Fig. 4, C, E, and G). In one of the other paired sets of samples, HAVOC partitions were reciprocally aligned with appropriate regions on different slides (Fig. 4, D, F, and H). The final case also highlighted distinct diffuse infiltrating biology exclusive to only one of the two slides (Patient Va_RED), which was again appropriately segmented by HAVOC (fig. S11). While quantification of regional cellularity provided further support for the proposed HAVOC groupings of the first two cases (Fig. 4), the third case pairing had a more uniform distribution of cellularity, further supporting that HAVOC likely uses a diversity of cytoarchitectural features to guide clustering (fig. S12). Together, these experiments highlighted the potential for HAVOC to appropriately map and align intratumor areas of heterogeneity across large tissue distances.

### HAVOC reveals distinct spatially separated differentiation states in high-grade gliomas

**Fig. 5.**
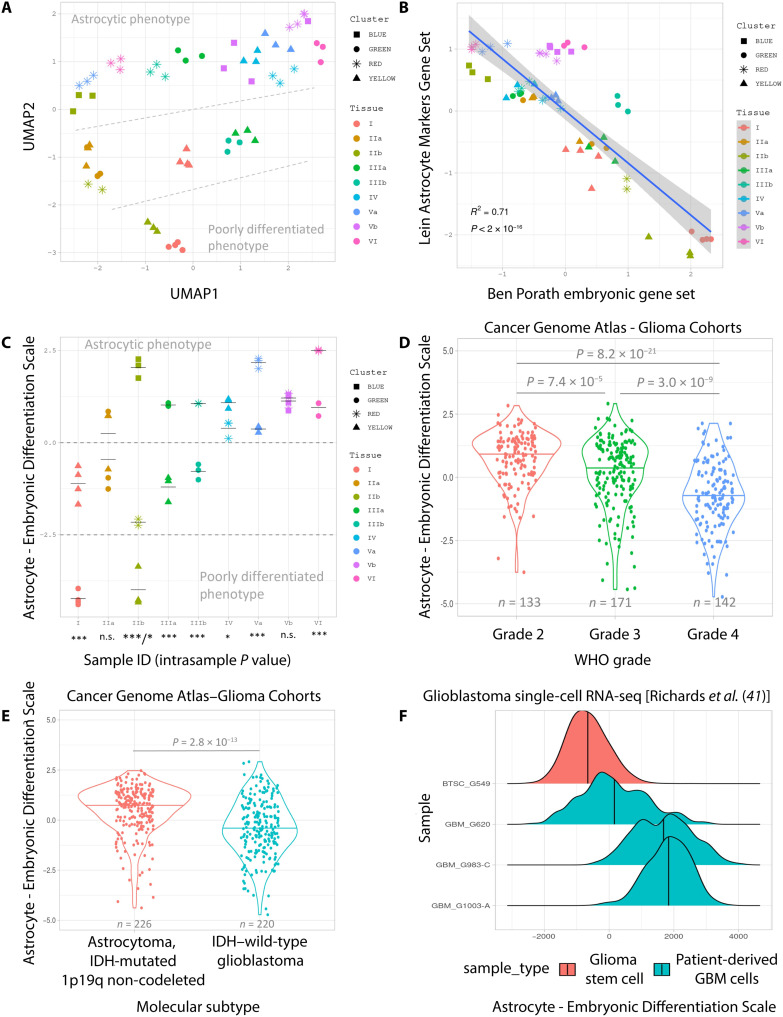
HAVOC reveals spatially organized patterns of molecular heterogeneity in high-grade gliomas. (**A**) Unsupervised analysis of 19 HAVOC-defined tumor regions across 6 high-grade gliomas by performing UMAP dimensional reduction of the ssGSEA scores of 64 proteogenomically concordant gene sets previously described in glioblastoma (*33*). (**B**) Inverse relationship between the ssGSEA scores of the embryonic stem cell state (Ben Porath ES_1) and the astrocytic differentiation gene set (Lein Astrocyte Markers Gene Set). *P* value generated by linear fit model. (**C**) Regional differences in state of differentiation (Astro-ES axis) across each of the spatially resolved and profiled slides. The level of statistical significance of the differences between the regions of each tissue slide was assessed by ANOVA; *P* values are indicated as follows: ***P < 0.001, **P < 0.01, and *P < 0.05; n.s., not significant. (**D** and **E**) Distribution of the Astro-ES axis in astrocytic tumors (non–1p19q-codeleted) from TCGA-GBM and TCGA-LGG cohorts stratified by WHO grade (D) and IDH status (E) (*P* values generated by ANOVA). (**F**) Varying distributions along the Astro-ES axis at the single-cell level in patient-derived GBM cells (*n* = 3) and glioma stem cells (*n* = 1); dataset previously published by Richards *et al.* (*41*).

Molecular descriptions of tumor biology are often defined based on sampling and profiling of a single tumor region (*39*). We therefore next wanted to examine whether HAVOC was detecting critical cellular states that may show reoccurring patterns of regional variation within different tumors. We therefore aggregated the profiles of the 19 regions spanning six IDH–wild-type high-grade gliomas (five WHO grade 4 and one WHO grade 3; concurrently profiled in this study) and carried out a group analysis (table S1). Using the previously defined set of 64 proteogenomically concordant molecular programs, unsupervised clustering of the 19 regions was partly driven by patient ID, speaking to the high intertumoral heterogeneity found in IDH–wild-type high-grade gliomas (fig. S13). This analysis also revealed spatial intratumoral variations in molecular programs in almost all profiled cases—including regional patterns of immune response, hypoxic response, proliferation, and embryonic differentiation states. Uniform manifold approximation and projection (UMAP) dimensionality reduction revealed a substantial influence of the later differentiation signatures across the cohort (Fig. 5A) [e.g., genes associated with an embryonic “poorly” differentiated cell state (*40*)]. This was further supported with a strong inverse correlation with regional programs associated with a mature astrocytic phenotype (*R*^2^ = 0.71, *P* < 2 × 10^−16^; linear fit model) (Fig. 5B). Supervised regional analysis of this astrocytic-embryonic differentiated axis found that at least five of the six profiled cases showed significant spatial differences (*P* < 0.005, ANOVA) (Fig. 5C). We found this high co-occurrence of these contrasting patterns notable, as tumors, across multiple organ sites, are often still approximated as being either “poorly” or “well” differentiated upon clinical presentation.

In TCGA, this differentiation axis correlated with aggressiveness, with both lower WHO grades (*P* < 7.4 × 10 ^−5^, *n* = 446) and IDH mutations (*P* = 2.8 × 10^−11^) of astrocytic (non–1p19q-codeleted) tumors showing significantly higher differentiation signal at the bulk level (Fig. 5, D and E). We also found support for both inter- and intratumoral variation along the astrocytic-to-embryonic axis in single-cell RNA data generated from both patient-derived glioblastoma tissue specimens and brain tumor stem cell cultures (BTSCs) (Fig. 5F and fig. S14) (*41*). While some samples analyzed showed complex expression patterns with multiple coexisting states of differentiation (GBM_G620; GBM_G983-C), others displayed fairly homogeneous signatures (GBM_G1003-A), presumably related to both the sampled region and the overall tumor biology. The notable spectrum of steady-state positions in differentiation states in expanded BTSCs suggests that generation of patient models from single individual tumor regions may only partially capture potential region-to-region variations in differentiation uncovered in this analysis. Together, these results highlight how neural network–guided multiregional sampling and profiling of cancer tissue can reveal previously unappreciated insights of biovariation that may be hidden and not immediately accessible from alternative single region profiling strategies.

### HAVOC biodiversity maps generalize to genomic differences and untrained tissue types

**Fig. 6.**
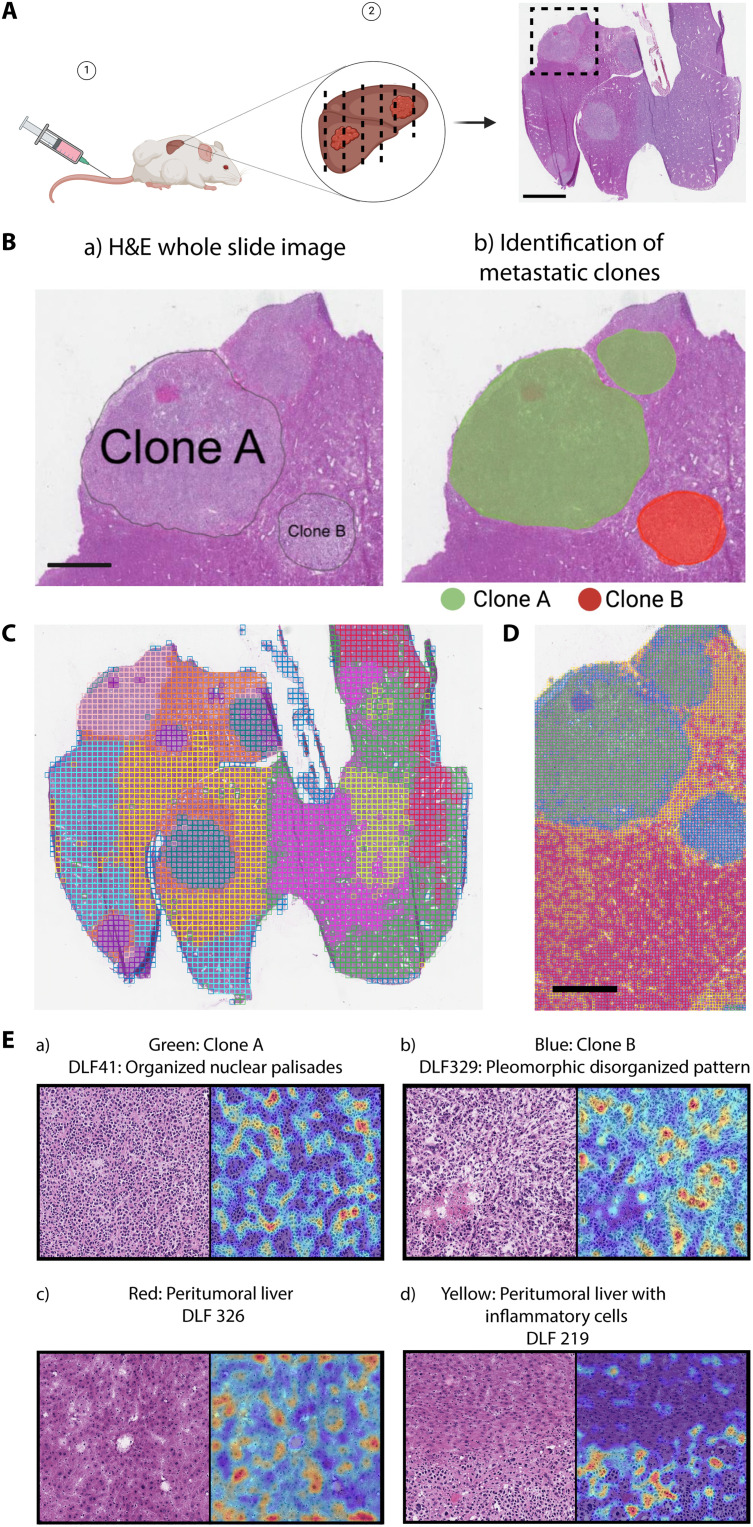
HAVOC defines genetically distinct metastatic clones. (**A**) Schematic and H&E-stained section of a mouse liver modeling polyclonal Kras^G12D/+^Trp53^−/−^ lung cancer metastases from Zhao *et al.* (*6*). Spatial profiling in the boxed region allowed benchmarking of HAVOC in this model. Scale bar, 5 mm. (**B**) Slide–DNA-seq and slide–RNA-seq of focused region provided ground truth of normal liver, tumor clone “A” and “B.” Black lines in the right panel indicate tumor boundaries. Scale bar, 2 mm. (**C**) HAVOC mapping of entire H&E sections with 11 partitions to ensure saturation of the different histomorphological patterns. HAVOC segmented all five tumors into stable groupings, identified in pink, teal, and purple. (**D**) HAVOC partitions (*k* = 4; tile width: 128 pixels) of the focused region mapped by slide–DNA-seq (original study) spatially divided tumor into two homogeneous subclones (green versus blue). Surrounding liver tissue was also separated into regions of peritumoral liver and immune infiltrates (red versus yellow) that match single-cell RNA-seq projection from the original study. Scale bar, 1 mm. Note: Color IDs of regions in (C) and (D) are unrelated due to the independent unsupervised nature of these analyses. (**E**) FAMs of selected differentially activated DLFs for each tumor region (a and b) and DLFs enriched in the peritumoral region highlighting liver regions with and without inflammatory cells (c and d). Note: DLF219 was enriched in peritumoral regions with inflammation and so is therefore specifically localized to the respective lower portion of the slide. Dimensions of image patches shown: 0.27 mm^2^.

As tumor heterogeneity is a challenge relevant to many cancer types (*42*), we next assessed the generalizability of HAVOC to other non-CNS neoplasms. First, we retrieved an available WSI of an experimental metastatic lung cancer mouse model in which two independent clones of a lung tumor were injected into the tail vein and subsequently resulted in multiple spatially distinct liver metastases (Fig. 6A) (*6*). Using spatial DNA and RNA-seq, two of the five metastatic deposits, in addition to the intervening liver tissue, were characterized in the originating study using copy number and expression pattern differences (Fig. 6B). Using HAVOC, we generated 11 total partitions (to ensure saturation of the different histomorphological patterns) and compared them with the characterized ground truth annotations. Image-based clustering segmented all five tumors into fairly homogeneous lesions, in addition to defining various regional histological patterns of the liver tissue (Fig. 6, C and D). Using the silhouette method, these five tumors formed two major clusters (*k* = 2 subclones) as the most parsimonious solution (fig. S15). Notably, HAVOC separated out both the two genomically distinct subclones and peritumoral liver tissue with and without inflammation, further supporting that distinct histomorphologic fingerprints can be leveraged to predict regions of potential genomic, transcriptomic, and cell composition heterogeneity (Fig. 6D). Subsequent FAMs exploring morphologic correlates highlighted distinct DLF-activating histomorphologic patterns within the tumor subclones defined by the original study. In clone A, FAMs highlighted a fairly advanced organization of tumor cells with abundant nuclear palisades, while clone B showed a more random arrangement with large, atypical cells scattered throughout the lesion. Furthermore, FAMs of the peritumoral liver tissue highlighted a differential distribution of inflammatory cells across HAVOC-proposed groupings, in agreement with the tumor, normal, and immune cell classes assigned from the original single-cell slide–RNA-seq projections (Fig. 6E).

While the other three remaining tumor regions were not genomically annotated in the original study (potentially due to cell throughput limitations of the spatial DNA sequencing technique), they did show similar histologic patterns to the more atypical tumor, confirming the ability of HAVOC to group metastases of similar histomorphologies, even when scattered across entire animal organs.

**Fig. 7.**
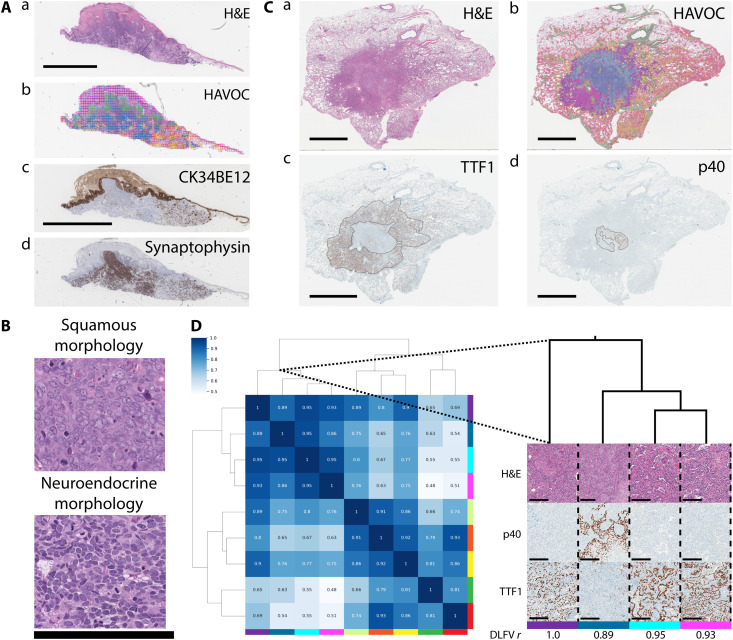
HAVOC generalizes to untrained tissue types. (**A**) (a) H&E-stained section of a dermatological “collision” tumor composed of distinct regions of squamous cell and neuroendocrine carcinoma. (b) HAVOC partitions resolved the distinct tumor types that matched the immunohistochemical ground truths. (c and d) CK34BE12 and synaptophysin highlight respective regions of squamous and neuroendocrine carcinoma. Scale bar, 2 mm. (**B**) Representative high-power magnification micrographs of HAVOC-defined distinct tumoral subclones highlighting the squamous (green partition) and neuroendocrine (blue partition) components. Scale bar, 200 μm. (**C**) (a) H&E-stained lung resection and (b) HAVOC map of a neoplasm showing divergent adenosquamous differentiation. Tumor subcomponents are highlighted with distinct (c) TTF1 (adenocarcinoma) and (d) p40 (squamous) immunohistochemical staining. HAVOC partition map showing subregions that align with the distinct tumoral patterns defined by the squamous/adenocarcinoma markers. Scale bars, 6 mm. (**D**) Hierarchical clustering of HAVOC-defined regions and representative high-power images of (a) H&E, (d) p40, and (c) TTF1-stained images reveals two distinct tumor subpatterns. The divergent p40^+^ squamous subregion has the lowest DLFV *r* value among these tumor regions. The colors under immunohistochemistry images correspond to the color codes of the HAVOC map (b). Scale bars, 200 μm.

To further test the generalizability of the HAVOC workflow to other untrained tissue types, we next applied it to “interesting” dermatopathology and pulmonary pathology cases showing a squamous neuroendocrine “collision” tumor and divergent adenosquamous tumor differentiation, respectively (Fig. 7). On the skin excision, the position of the squamous cell carcinoma (in situ) is highlighted by the high molecular weight keratin (CK34BE12). Within the dermis, a distinct infiltrative neuroendocrine neoplasm composed of sheets, nests, and cords of round basaloid cells was labeled with synaptophysin. HAVOC-proposed groupings of this specimen distinguished the distinct squamous and neuroendocrine morphologies in alignment with the distinct immunohistochemical staining (Fig. 7, A and B). HAVOC partitioning of the case of adenosquamous carcinoma also showed a high spatial concordance to TTF1 (adenocarinoma) and p40 (squamous carcinoma) immunopositive tumoral components (Fig. 7, C and D, and fig. S16). Together, these data demonstrate that HAVOC can serve as a tissue type– and molecular-agnostic tool to map biodiversity in different cancers.

## DISCUSSION

Intratumoral heterogeneity has emerged as a key concept in current precision medicine efforts (*42*). While there have been technological breakthroughs to map such spatial biodiversity at the genomic, transcriptomic, and proteomic level, the relative discord between the upper limits of tissue profiling throughput and tumor sizes creates an important bottleneck for comprehensive analysis of most tumor specimens (*2*, *4*, *7*). Here, we highlight how a neural network–based tool (HAVOC) can detect and quantify spatial distributions of cancer biodiversity not only across individual sections but also on larger tumor specimens in a hypothesis-agnostic manner. We go to great lengths to prospectively validate that these proposed histomorphologic patterns of spatial heterogeneity correlate with human interpretations, key biomarkers of aggressiveness, and global genetic, transcriptomic, and protein-level differences. Using this tool, we partitioned and profiled 19 distinct regions from 6 IDH–wild-type high-grade glioma specimens and uncovered that different spatially confined states of differentiation can coexist within the same tumor. While the defined poorly differentiated state correlated with many important parameters of clinical aggressiveness (WHO grade and IDH status), it does not appear to mirror with proliferation or tumor-initiating potential in previous studies, adding another layer of complexity to existing models of tumor heterogeneity. Such findings also underscore the need for histomic mapping to help guide and improve representative sampling and characterization of heterogeneity across entire tumor resection specimens. While we focused our discussion and validation efforts on spatial patterns of differentiation, there were many additional case-by-case geographic differences identified that were outside the scope of this study (e.g., proliferation, hypoxia, and immune response). We therefore make this unique morphology-driven spatial analysis of the glioma proteome available for further exploration through an interactive data portal [Brain Protein Atlas (*29*); https://www.brainproteinatlas.org/dash/apps/ad].

Despite effectiveness at resolving intrasample variation in our glioma cohort, we suspect that a high degree of interpatient variation among the selected cases precluded establishment of robust morphomolecular relationships across multiple independent samples. For example, while some gliomas’ major axis of intratumoral variation was along the differentiation axis, other tumors’ morphologically variable regions were dominated by heterogeneity in hypoxia and/or immune response (fig. S13). While there may be some robust associations that are transferable across cases, this will likely require extending HAVOC to guide profiling of much larger cohorts.

This aspect brings important distinctions to how HAVOC complements other computational approaches to mapping intratumoral molecular heterogeneity directly from histology. Approaches that aim at directly training and/or correlating deep learning outputs to specific molecular events are inherently empirical and may be most effective at predicting fairly robust molecular signatures that are driven by heterogeneous cellular compositions (e.g., presence of lymphocytes and vessels) and/or a handful of transcriptional profiles (e.g., cell division) (*22*, *23*). While these may be highly effective and cost-efficient in some clinical and research contexts, as discussed above, such approaches may not have the capacity to extend and generalize across all cancer types and relevant molecular pathways of interest. This presents an important limitation and gap for targetable biological programs that may not have strong “histomolecular” correlates, for cancers with a high degree of heterogeneity, and for more modern personalized medicine strategies (*27*, *28*). A more hypothesis-free approach has been used with supervised deep learning approaches to identify specific patterns within tumors (e.g., areas devoid of tumor-infiltrating lymphocytes and stromal elements) that may signal differential biology (*14*). These “at risk” regions provide spatial targets for prospective and personalized profiling of individual cancer specimens. We believe that HAVOC extends this key concept further by decoupling the need for any predefined feature that may potentially bias or limit exploration into mapping of biovariation with associated phenotypic changes. While, in certain circumstances, this may compromise the sensitivity for detecting subtle spatial variations in important molecular programs, we believe that it provides a highly dynamic and generalizable solution for personalized discovery and characterization of spatial changes in tumor biology. It therefore can be directly extended not only to a variety of tumor types but also to the longitudinal analysis of cancer in which molecular patterns may be influenced by both tumor progression and treatment-related effects (*9*).

HAVOC does not require any dedicated tissue or substantial computational resources and contains only a handful of tunable parameters (e.g., tile size and cluster numbers; elaborated on in Materials and Methods) that can be easily adjusted depending on the specific context (e.g., smaller tumor deposits). These capabilities, when combined with the processing of serial sections for molecular analysis, provide a computational approach for the characterization of intratumoral heterogeneity at practically any scale and level of resource availability. Moreover, as we demonstrated in this study, the large diversity of images used to train the default CNN within HAVOC provides an adaptable unsupervised framework to detect tumor heterogeneity in a relatively species-, tissue-, molecular platform–agnostic manner. We, therefore, envision HAVOC to serve as a routine and powerful research tool for mapping intratumoral heterogeneity across both large-scale and more intricate (*n*-of-1) tissue profiling studies. Given its ease of implementation and compatibility with formalin-fixed paraffin-embedded (FFPE) sections, we envision HAVOC becoming an essential tool to map heterogeneity on all clinical and research tissue-based studies to help provide more systematic approaches to tissue selection for ongoing personalized precision medicine efforts.

HAVOC’s effectiveness at detecting histomorphologic pattern differences means that it can be theoretically extended to nonneoplastic and immunohistochemistry-resolved tissue heterogeneity that may be niche and epigenetically patterned and independent of genetic alternations (e.g., copy number). Moreover, when coupled with salient feature activation mapping, it can complement spatial transcriptomic and proteomic approaches by providing phenotypic correlates (e.g., edema and increased nuclear:cytoplasmic ratio) to explain/predict interesting expression-level differences. To facilitate adoption, we have packaged HAVOC in a number of ways to promote ease of use. For large-scale initiatives, we provide source code to allow HAVOC to be deployed locally on large cohorts in an automated manner. For more translational researchers and clinicians, we also host HAVOC in a cloud-based server (https://www.codido.co) that allows analysis of digitized slides (.SVS/.JPG/.NDPI) without any need for advanced software expertise or hardware.

There are some important caveats of HAVOC that should be considered when using this tool. First, its fundamental dependence on tissue patterns means that it can be influenced by nonbiological factors (e.g., tissue folds, tears, focus, suboptimal uneven staining, and tissue edges). In our experience, we found that these artifacts can easily be identified and excluded from downstream analysis, both by careful post hoc analysis or by supervised classification of clusters to label tumor-specific partitions. Coupling HAVOC partitions with automated cellularity metrics (Figs. 1F and 2H) or other pertinent features of interest could also help potentially improve the predictability and interpretability for large complex specimens. Last, the sensitivity of HAVOC for very subtle and intermixed patterns of biodiversity likely also needs to be assessed in a context-specific manner. These biological factors may dictate the particular type of molecular profiling technique best suited for HAVOC pairing. In summary, HAVOC provides a highly flexible, generalizable, accessible, and scalable approach for mapping histomorphologic-correlated phenotypes and could serve as an essential tool to explore and document biologic heterogeneity in human tissue and disease.

## MATERIALS AND METHODS

### Ethics statement

The University Health Network Research Ethics Board (UHN REB) approved the study REB #17-6193 as it has been found to comply with relevant research ethics guidelines, as well as the Ontario Personal Health Information Protection Act (PHIPA), 2004. Patient consent was not directly obtained, and instead, a consent waiver for this study was granted by UHN REB as the research was deemed to involve no more than minimal risk as it used exclusively archival tissue specimens.

### Tissue cohort development and digital scanning

All clinical cases included in our study cohort were retrieved from UHN archival tissue specimens. To assess potential associations between DLFV correlation coefficients and human-perceived differences, an initial brain tumor tissue cohort (*n* = 40) was developed that largely consisted of diffuse glioma cases (IDH wild type and IDH mutants, WHO grades 2 to 4). To compare proteome-level programs between distinct partitions, cases were further examined to define regions relatively pure in tumor content (e.g., not regions of low tumor purity) and with partitions sufficiently large enough for LCM and LC-MS/MS analysis. In total, the LC-MS/MS cohort included seven high-grade gliomas (*31*). Six cases had glioblastoma histology and an IDH–wild-type immunohistochemical staining pattern. The final case was an IDH–wild-type high-grade glioma with a WHO grade 3 histology (table S1). Additional independent confirmatory cases were also selected, which displayed region-to-region heterogeneity in their MIB1/Ki-67 proliferation indices (*n* = 5 independent cases). The collision tumor in the skin and the lung adenosquamous carcinoma was also retrieved from our local cancer center (UHN). The H&E section of the metastatic lung cancer model was provided directly from the authors of the relevant study (*6*). The entire generated local cohort was digitally scanned as WSIs with a compression quality of 0.70 and a magnification of ×20 on a Leica Aperio AT2 scanner.

### Implementation of the deep CNN

We used a previously trained pathology-optimized version (*32*) of the VGG19 CNN (*43*) to extract the DLFVs used during HAVOC partitioning (*30*). Specifically, the original VGG19 model was optimized using transfer learning on a set of 838,644 pathologist-annotated image patches spanning over 70 brain tissue and tumor types derived from more than 1000 patients (*32*). We previously showed that this training diversity led to fine-tuning within the network that aligned with human-discernible histopathological feature representations that are applicable across multiple malignancies (*15*, *32*). Given the unsupervised nature of HAVOC, the CNN was used without having to undergo any additional training or optimization for this application. Previously learned class labels from the original fine-tuning of the VGG19 were decoupled from this analysis, and instead, only the feature representations, in the form of a 512-dimentional vector from the global average pooling layer of the CNN, before SoftMax reduction and classification, were extracted and used for image-based clustering (*32*).

To highlight potential relationships between outputs and the specific CNN used, we compared HAVOC heterogeneity maps generated using the VGG19 CNN with and without pathology-focused transfer learning (fig. S17). While both classifiers did perform well and relatively similarly at demarking major boundaries of tissue variation, we found that the classifier that benefited from domain-specific transfer learning was more specific at differentiating normal from neoplastic tissue regions in both primary brain and nonbrain tumors. In most of the scenarios we examined, intratumoral subpopulations were also more effectively defined by the fine-tuned version, with the “out-of-the-box” VGG19 CNN requiring additional subclusters (high *k* values) to define regional biovariation in both the brain and nonbrain tumor specimens (fig. S17).

Therefore, while diversely trained CNNs, even outside of pathology, may serve as generalizable feature extractors, this analysis does support that some degree of domain-related fine-tuning may help prioritize and triage specific histologic patterns over others and provide slightly different results, some with potentially critical biological implications. Given the “black box” nature of the extracted features, specifications of the CNN used and fine-tuning steps are likely parameters that will need to be indicated in future applications for standardization and reproducibility.

### Regional partitioning and estimation of heterogeneity

Tissue partitions by HAVOC were generated using an unsupervised image clustering framework as previously described (*15*). Briefly, WSIs for each clinical specimen of interest are first tiled into individual 0.066- to 0.27-mm^2^ image patches. These values correspond to a patch length of 258 μm (512 pixels) and 517 μm (1024 pixels) and represent the approximate field of view diameter of a 40× to 100× objective on a standard modern microscope. We found that this magnification offers a favorable magnification that can detect both individual cellular differences (e.g., nuclear features) and more complex secondary tumor structures (e.g., infiltration and glands/rosette formation) that help define diverse tumor types and patterns of differentiation. We do, however, realize that, for some contexts, smaller or larger tile sizes can be preferred to highlight specific high- or low-power features of interest, respectively, and/or computational constraints of the user. Thus, we have left the tile size completely tunable in HAVOC along with the number of partitions. To highlight this relationship, we generate and provide HAVOC maps for a representative example of a demonstrative WSI from an epilepsy case (fig. S18). By varying the tile size from 4096, 1024, 256, 128, and 64 pixels (width), we found a trade-off of resolution at larger tile sizes with increased noise at smaller tile sizes. It is also noteworthy that smaller tile sizes exponentially increase the computational demand of the workflow and took much longer to generate the heterogeneity maps. The particular size of the image patches used in this study was also largely dictated by the size of the available tissue (smaller tiles for smaller tissue) and histomorphologic pattern of interest [e.g., cytoarchitecture versus nuclear patterns benefiting from larger (low-power magnification) versus smaller (high-power magnification) tiles, respectively].

Histomorphologic fingerprints for each tile are then generated by averaging the DLF values from the final global average pooling layer of the VGG19 CNN. We refer to these 512 feature representations as the DLFV. As this network was tuned on a diverse set of histological images, many individual DLFs align with human-identifiable histologic features, including fibrosis, epithelium, and mucoid patterns, thus allowing the network to group image patches, with relatively similar morphologies, into meaningful clusters (*32*). To identify spatial transitions in histomorphological patterns for each case, the DLFVs generated from each tile are first scaled feature-wise and then are hierarchically clustered, using Ward’s method, as previously described (*32*). Images that group together by specific clustering thresholds (default *k* = 9) are deemed to show a relatively similar histomorphologic signature and are grouped together to form a HAVOC partition. In our experience, these often tend to show spatial relationships concordant with our histopathologic review. The relative distances (similarity) of each HAVOC partition (spatial region) are quantified using the Pearson correlation coefficient of each region’s average DLFVs and also tended to quantitatively correlate with histomorphologic differences perceived by humans.

### Region-specific DLF selection and mapping

To understand which morphologic features were potentially driving, or at the very least associated with, the unsupervised slide subgroupings, we first identified the most significantly enriched DLFs in each of the HAVOC-proposed groupings. To visually determine what morphology such DLFs were detecting, we assessed image tiles at the extremes of the DLFs of interest across the entire slide (both the highest-scoring and the lowest-scoring image patches). Interpretations of potential morphological differences between the tiles at the two extremes were provided by two or more blinded members of our team. This qualitative assessment was then complimented and validated by generating isolated FAMs of the candidate DLFs of interest to evaluate whether the activated tile coordinates matched the location of the histologic feature suggested. Python code used to generate the FAMs can be found at BitBucket (https://bitbucket.org/diamandislabii/faust-feature-vectors-2019).

We note that selection of FAMs with a “punctate” pattern of staining for these analyses was intentional as we find that they tend to allow for a more objective assessment of what potential histological features may be correlated with/driving the HAVOC partitions. For example, the punctate nature of FAM219 in Fig. 6Ed appeared to avoid the larger liver parenchymal cells in the lower part of the image, suggesting that it might be more specific for the inflammatory cell reaction found in this region and not color differences in the surrounding liver parenchyma. A more homogeneous FAM would perhaps make it more difficult to interpret as it would make it challenging to align with the histological features proposed by our team.

To highlight the variability of correlated DLFs and FAMs from one another, we provide multiple representative FAMs from features we highlighted to be co-enriched in the RED region of the WSI presented in Fig. 2 (see fig. S8). This highlights how different correlated FAMs can localize to both similar (e.g., 442 versus 503) and distinct (e.g., 442 versus 118) regions of a particular field. The specific absence of labeling in the region containing a large vessel provides support that the chosen features are “tumor” specific and not nonspecifically labeling artifacts from one region to another. It is important to note that because many of the FAMs are chosen to be enriched in one of multiple related tumor/tissue regions, as compared to independent tumors/tissue types, a truly homogeneous activation in all tumor cells is also less likely in these types of analyses. Therefore, we find that punctate patterns focused on more complex subtle differences between the related tumor regions represent the most informative pattern of intratumoral regional DLF FAMs.

To supplement individual DLF FAM analysis, we also provide representative plots of the cumulative explained variance of principal components (PCs) for one central nervous and one non-CNS tumor (fig. S19). For these cases, we found that 21 and 25 PCs were needed to capture 80% of the total variance in tissue patterns (fig. S19, A and B). As expected, many of the top PCs appear to localize DLF signature variance to the most cellular tumor regions (fig. S19, C to E). There are, however, subtle spatial differences in the activations of individual PCs, suggesting that even different PCs may be capturing distinct but relevant nuclear features to resolve different tissue patterns (adenocarcinoma versus squamous cell carcinoma) among tumor areas (fig. S19, D, F, and G).

### Histomorphologic evaluation of HAVOC-defined heterogeneity

Both computational and human evaluation approaches were used to evaluate the histopathologic correlations of HAVOC-proposed regions of heterogeneity and determine the optimal number of WSI partitions when appropriate. In most scenarios, we found that computational metrics (e.g., Gap Statistic, Silhouette Coefficient, Elbow, and Calinski-Harabasz Index) to compute the optimal number of partitions of WSIs was not particularly robust for differentiating clinically relevant histologic pattern variations as they were heavily influenced by the overall number and size of distinct histomorphologic patterns found within WSIs. While they each performed well in some cases, they often led to solutions that either overestimated (high *k* solution) or underestimated (low *k* solution) the degree of histological heterogeneity that was objectively discernible by our team. As an alternative solution, we therefore empirically defined a default *k* value that would give more consistent results. We did this by examining the added benefit of each additional partition from *k* = 2 to 10. As each successive cluster often divided increasingly more subtle histological differences than the previous one, we saw progressively higher correlation scores across the regions with higher *k* values (highlighted in fig. S3). For most cases, we also found that the vast degree of pathologist-discernible patterns of histomorphologic variations reached saturation after seven to eight WSI partitions irrespective of the overall level of complexity and tissue heterogeneity. To empirically link these observations, we developed a semiquantitative heterogeneity score (severe, moderate, and mild) by examining the paired tissue patterns found across numerous HAVOC partitions (*n* = 40 WSIs, *n* = 200 total comparisons; tile size = 1024 × 1024 pixels, *k* = 9). Specifically, paired permutations of histologic regions delineated in HAVOC maps (e.g., Fig. 1E) were assigned a heterogeneity score of severe when tumor partitions showed obvious pattern differences, even at low magnifications (5× to 10×) (e.g., purple versus cyan in Fig. 1E). A moderate classification was reserved for tumor partitions showing obvious pattern differences only at high magnifications (20× to 40×) (e.g., cyan versus blue). Last, a mild score was assigned when tumor partitions showed only subtle and/or subjective pattern differences, even at higher magnification. We also carried out a comparison of tumor and normal tissue as a control (most severe pattern differences) (e.g., green/yellow versus blue). We provide an additional glioma case as a demonstrative example of this scoring system (fig. S20).

Once these evaluations were completed, we plotted these consensus ratings (*x* axis) with the corresponding DLFV correlations (*y* axis) for these partition comparisons (e.g., Fig. 1H) to generate Fig. 1I. Because there were often multiple valid comparisons for each slide, we included all available data for comprehensiveness. In total, this resulted in 200 overall comparisons over 40 WSIs. Overall, we assigned moderate heterogeneity as the most subtle form of pathologist-discernible patterns of histomorphologic variation that could be reliably/objectively resolved. The average DLFV *r* for these regional pairs corresponded to a value of 0.75. As successive HAVOC partitions tended to define tissue regions with smaller and smaller histological differences (and also an overall higher global paired DLFV *r* value), we found that after ~7 to 8 WSI partitions, we could reliably resolve the vast majority of the overall tissue complexity found in brain tumor specimens (figs. S1 to S3).

We note that while seven partitions worked well for mapping most histomorphologic patterns of variation in our clinical cohort, the clustering framework is easily tunable and can be extended to additional partitions using a stepwise *k*-means clustering approach to explore additional and more subtle regions of heterogeneity in complex tissue specimens (figs. S1 and S16). To capture finer microscopic details, relevant in some circumstances, the size of the image patches can also be tuned to the appropriate application (fig. S18). For examination of relatively large regions of cellular tumor, image patches with a width of 512 and 1024 pixels worked extremely well and reliably in our experiences (e.g., Fig. 2). For more focal deposits (e.g., Fig. 6), the tile size can be reduced to 256 or 512 pixels in length to minimize intrapatch pattern variation from edges and other positional artifacts.

### LCM and LC-MS/MS proteomic profiling

Tissue sections were stained before LCM to improve contrast and provide references across the slides to guide precise microdissection as previously described (*33*, *44*). Specifically, FFPE tissue blocks were sectioned (at 10 μm thickness) and mounted onto Leica PEN slides (catalog no. 11505189). Slides were subsequently deparaffinized with 100% xylene (2×), 100% ethanol, 95% ethanol, 70% ethanol, and 50% ethanol (3 min each). These slides were then stained with hematoxylin (1 min), rinsed in deionized water (1 min), and stained in 1% eosin Y (Fisher Scientific). Slides were finally air-dried before LCM.

Regions of interest from these sections were microdissected using Leica LMD 70000 (Leica Microsystems Inc., Bannockburn, IL). HAVOC-generated color tiled maps were used as a reference to guide parameters for dissection. Samples were collected in an Eppendorf tube and subsequently stored at room temperature until further sample preparation began.

Proteomic extraction was performed with the addition of 50 μl of 1% Rapigest and 200 μl of dithiothreitol, ammonium bicarbonate (50 mM), and tris-HCl solution to each sample. Samples were subsequently sonicated using Bioruptor Plus on high with 30-s intervals for 1 hour. Solutions were heated to 95°C for 45 min, followed by 80°C for 90 min with a ThermoMixer. Twenty microliters of iodoacetamide was then added to each solution in the absence of light for alkylation. One microgram of trypsin/Lys-C mix was then added to each sample and reacted overnight at 37°C. The solutions were subsequently acidified with trifluoroacetic acid at a final concentration of 1% ahead of stagetip cleanup.

In preparation for MS analysis, samples were desalted using Omix C18 tips following the manufacturer’s protocol. Elution of peptides was completed with 3 μl (0.1% formic acid, 65% acetonitrile), and dilution of peptides was completed with 57 μl (0.1% formic acid in MS water). Eighteen microliters of solution (2.5 μg of peptides) was loaded with an autosampler for MS as previously described (*33*). Briefly, peptide elutions from EASY-Spray column ES803A occurred at a rate of 300 nl/min with increasing concentration of 0.1% formic acid in acetonitrile over a 1-hour gradient. This setup was coupled to Q Exactive HF-X with a spray voltage of 2 kV and with a 60-min data-dependent acquisition method. The full MS1 scan was completed from 400 to 1500 mass-charge ratio (*m*/*z*) at a resolution of 70,000 in profile mode, with the top 28 ions selected for further fragmentation with a higher energy collisional dissociation (HCD) cell. Fragment detection occurred with an Orbitrap using centroid mode set at a resolution of 17,500. The following MS parameters were used: MS1 automatic gain control (AGC) target was set at 3 × 10^6^ with a maximum injection time of 100 ms; MS2 AGC target was set at 5 × 10^4^ with a maximum injection time of 50 ms, an isolation window of 1.6 Da, an underfill ratio of 2%, an intensity threshold of 2 × 10^4^, and a normalized collision energy of 27; charge exclusion was set to fragment 2+, 3+, and 4+ charge state ions only; peptide match was set to preferred; and dynamic exclusion was set to 42 (for 90-min method).

### Multislide tumor histomic profiling and organization

HAVOC evaluation of larger and even entire tumor specimens was completed by histomic profiling of each H&E-stained section for each of the corresponding tumor blocks. Unsupervised analysis of each WSI was completed individually as described in previous sections and then merged in a separate and final step. For the example, of the 12-slide tumor specimen shown in Fig. 3, the number of clusters for each WSI was set to 7, within the range that results in saturation of perceivable histomorphologic patterns at a tile size of 0.27 mm^2^. Members of our team reviewed the 84 HAVOC partitions (7 per WSI) and provided annotations into one of the seven major annotations that were felt to be well represented in this case. These labels were meant to help benchmark how well similar regions, found on different slides, could be grouped together using their DLFV histomic fingerprints. To do this, the average DLFVs of HAVOC partitions from the related WSIs of the specimen were then subsequently organized together in an 84 × 84 matrix of pairwise Pearson correlation coefficients using hierarchical clustering. The “Histology” track showed the provided human annotations for each of the 84 regions.

While we attempted a number of validation metrics (e.g., Gap Statistic, Silhouette Coefficient, Elbow, and Calinski-Harabasz Index) for computing the optimal number of both slide- and specimen-level HAVOC clusters, we found that the best performer substantially varied from context to context and did not always offer the best demarcation of pathologically important subclusters. For example, a large amount of normal tissue on a slide could skew the optimal clustering solutions to differentiate only tumor from normal tissue (e.g., the WSI presented in Fig. 1) and under-call more subtle but relevant subpopulations. Similarly, fragmented tissue elements sometimes led to many slight variations in relatively homogeneous regions and overclustering solutions.

Because of these numerous external variables, we found that incorporating some judgment was necessary when choosing the optimal cluster solution that best reflects the biology of each sample. To objectively do this, we used the dendrogram pattern generated by the agglomerative hierarchical clustering of DLFV *r*’s between HAVOC partitions and compared it to multiple consensus groupings of overall histologic impressions (fig. S10). We then objectively tested and validated these impressions statistically (table S3). We provide a separate script for this final proposed WSI merging step in the associated repository. Multislide Pearson correlation matrices of HAVOC-proposed groupings were generated using Matplotlib and Seaborn Python data visualization libraries.

In addition to this qualitative validation strategy, evaluation of the fidelity of the multislide regional alignment for patients with multiple related slides/tissue sections included more objective metrics such as regionally restricted immunohistochemical staining patterns (e.g., Ki-67, CK4BE12, and synaptophysin; Figs. 3C and 7), cell counts (e.g., fig. S12), and LC-MS/MS analysis (e.g., Fig. 4 and fig. S11).

We also emphasize that there are multiple valid approaches to mapping heterogeneity across multiple slides. For example, all tiles across multiple WSIs could be merged in the initial step and cluster once (“tile-level global analysis”) rather than separately clustering regions on individual WSIs and merging groups in a second separate post hoc step (“WSI-level global analysis”). Conceptually, we felt that stratifying HAVOC clusters equally across individual WSIs, before merging related slides, reduced the complexity of downstream benchmarking exercises (see below). It also likely increases the sensitivity of resolving focal slide-specific patterns that might not be well represented across multiple slides or large regions of the entire specimen. Combining multiple WSIs into a single unified tile-based analysis may alternatively provide more sensitivity at finding subtle global similarities across multiple WSIs. Overall, we believe that both strategies are valid and, at the appropriate overall partition number and tile sizes, likely give fairly similar results. We found, however, that HAVOC maps can substantially diverge when the optimal partition number is unclear. This is likely heavily dependent on the number of valid tissue patterns across individual WSIs and/or entire tissue specimens, which may markedly vary from specimen to specimen.

Because of this variable case-to-case tissue complexity, one of the major motivations of choosing the presented strategy is that it allowed us to maintain the empirically determined optimal number of HAVOC partitions at the slide level (e.g., ~7 HAVOC partitions/WSI; fig. S3), which we found to be more reliable than purely computational approaches. As a result, we felt that this was the more natural approach to present multi-WSI analysis as it extended the existing workflow without needing to recalibrate optimal partition numbers for larger specimens.

While it is difficult to authoritatively compare similarities and differences between the approaches across all possible scenarios in this proof-of-concept study, we provide a simplified demonstrative example of three related WSIs from a large epilepsy resection specimen to highlight the variable strategies (fig. S21). In the WSI-level global analysis, we partition each WSI into three HAVOC regions (total: nine HAVOC regions over three slides). In the tile-level global analysis, we also distribute the totality of tiles across the three WSIs into nine HAVOC regions for comparison. Overall, the correlation matrices show that for both approaches, the major patterns of substantial biovariation are in the separation of gray and white matter. Even with this simple example, the relative intermixing of the subregions in the tile-level global analysis (*k* = 9) would have created critical technical limitations in our HAVOC validation strategies.

First, when benchmarking HAVOC with respect to human histological interpretations (Fig. 3), we found it relatively easy to ask our team members to describe HAVOC partitions on individual WSIs, as relative regional variation in tissue patterns could be leveraged as “on-slide” comparisons without the need to go back and forth between WSIs for annotation. We felt that this exercise would become progressively more challenging and subjective if scattered tiles belonging to a specific cluster are distributed across different WSIs. Second, by combining WSI-level interpretations with a final and independent multislide clustering step, we felt that we were removing some of the subjectivity in assessing performance that would be lost if the overall “optimal” *k* value and interpretations of resulting partitions were done completely respectively in a single step (e.g., tile-level global analysis). In addition, for the complementary regional molecular/immunohistochemical analyses (e.g., Fig. 4), the potential for more complex subpatterns generated by the tile-level global analysis could have also introduced more subjectivity and challenges to the microdissection/analytical steps used. With these initial proof-of-concept validation efforts completed and the aforementioned considerations in mind, we believe that both approaches can be applied to future cases.

### Statistical analysis

MaxQuant Andromeda (v1.5.5.1) search engine was used to process MS raw data files against the Human SwissProt protein database (July 2019 version). Proteins were filtered to include only those appearing at least 60% within a sample. Raw protein values were log_2_-transformed, with nonvalid values imputed (downshift = 0.3, width = 1.8). Activation of proteomic programs at gene set level was analyzed by single-sample gene set enrichment analysis (ssGSEA) (*45*) using the GSVA Bioconductor package (https://bioconductor.org/packages/release/bioc/html/GSVA.html), and psSubpathway (*46*) was used to establish the profiles of gene sets characterizing different tissue regions (Fig. 2D). ssGSEA was used to define pathways enriched in each HAVOC-proposed region. One-way ANOVA testing and Tukey’s post hoc test were completed in R (v4.0.4) to identify differences in enriched pathways across HAVOC-proposed groupings. Student’s *t* tests were used to test the difference in Ki-67 proliferation indices across HAVOC-proposed groupings and other focused analyses as indicated. Additional statistical tests used for individual analyses are mentioned in the appropriate text and figure coordinates.

### Gene set enrichment analysis

GSEA was used to understand the biological significance of the regionally distinct molecular profiles both on individual samples (Fig. 2) and at a cohort level (Fig. 5). Three gene expression datasets were analyzed: (i) proteomics dataset generated in this study. For this, proteins with >25% of missing values were removed, resulting in a dataset of label free quantification (LFQ) intensity values with 1920 proteins across 60 injected aliquots, which typically included three technical replicates per tissue specimen. (ii) RNA-seq data and associated clinical information were from the merged cohort of LGG and GBM (*47*) retrieved from cBioportal (http://www.cbioportal.org/study/summary?id=lgggbm_tcga_pub). Oligodendrogliomas were excluded from the analysis, resulting in a cohort of 446 diffuse “astrocytic” tumors. (iii) The final cohort represented normalized single-cell RNA data previously published by Richards *et al.* (*41*). This dataset was downloaded from the Broad Institute’s Single Cell Portal (https://singlecell.broadinstitute.org/single_cell/study/SCP503). To reduce signature enrichment bias due to poor sequencing coverage, samples with less than 10,000 unique molecular identifiers were excluded from the analysis, and the resulting dataset contained 28 glioma stem cell samples and 6 patient-derived GBM samples.

The gene expression profiling was focused on a list of 64 gene sets previously described by our group (*33*), which were selected from the MSigDB-7.2 database (https://www.gsea-msigdb.org/gsea/msigdb) on the basis of being informative in glioblastoma and showing a high degree of proteotranscriptomic correlation. Here, we used a revised list of 64 gene sets that differs from the previously published one as follows: (i) The pathway “REACTOME_HSP90_CHAPERONE_CYCLE_FOR_STEROID_…” was dropped during the version upgrade to MsigDB-7.4, and (ii) the signature LEIN_ASTROCYTOMA_MARKERS was included as a result of screening for signatures informative of astrocytic differentiation. ssGSEA was performed with the Bioconductor package GSVAv1.44.2.

The developed Astrocyte-Embryonic Differentiation (Astro-ES) axis is a composite score designed to cover the spectrum of cellular differentiation that is found in diffuse gliomas, where high values correspond to a “well differentiated” astrocytic phenotype and lower values with an embryonic “poorly differentiated” state. This score is calculated by zero-centering the ssGSEA scores of the LEIN_ASTROCYTOMA_MARKERS and BENPORATH_ES_1 gene sets and then subtracting the latter from the former.

### Estimation of regional cellular density in HAVOC-defined regions

We estimated cell density in profiled regions to complement the interpretation of HAVOC-defined regions using various digital quantification approaches. Where specified, WSI data files (.svs format) were loaded on QuPath v0.3.2. For each region of interest, five circular sampling areas spanning 100 to 125 μm^2^ and histological representative of the region of interest were delineated and followed by running Estimate Stain Vectors and Cell Detection Tool with default parameters. The density of each sampling area was computed as follows: number of detections × 100/area, and finally, the regional cellular density is the average of the cellular density of the five sampling areas. Last, the regional cellular density was categorized according to the following arbitrarily selected reference framework: ultrahigh (>1.2), high (8 to 1.2), mid (6 to 8), and low (<6).

In later analyses, we also incorporated a companion automated deep learning approach for quantification of cellular density across multiple HAVOC partitions less reliant on manual delineation of regions of interest. Briefly, we optimized Mask R-CNN (*48*) using the Detectron2 library to serve as a nuclear instance segmentation tool similar to what we previously described (*49*). We extracted 128 × 128 pixel image patches from different WSIs containing approximately 20 to 30 cells per patch. These were loaded into a web-based annotation tool (www.makesense.ai) to generate training examples of various nuclear cell shapes. Nuclei in these training images were manually segmented and labeled by our team, and these manually annotated image patches were used to train a Mask R-CNN model. To do this, we used transfer learning to fine-tune the ResNeXt-101 R-CNN model, which was pretrained on the COCO 2017 dataset (~37 COCO epochs). We provide representative examples of the Mask R-CNN annotations when it was used to highlight sensitivity and specificity of this tool. ANOVA and *t* testing were used to compare cellularity counts (per 128 × 128 pixel patches) between different regions.
