# Supplementary material for: HAVOC: Small-scale histomic mapping of cancer biodiversity across large tissue distances using deep neural networks
Source: Sci Adv. 2023 Sep 29;9(39):eadg1894. doi: 10.1126/sciadv.adg1894 (PMC10541015; doi:10.1126/sciadv.adg1894)
Supplement: Supplementary file 1 — Figs. S1 to S21 Tables S1 to S3 [file sciadv.adg1894_sm.pdf]

Science Advances
AAAS

Supplementary Materials for

HAVOC: Small-scale histomic mapping of cancer biodiversity across large tissue distances using deep neural networks

Anglin Dent *et al.*

Corresponding author: Phedias Diamandis, p.diamandis@mail.utoronto.ca

Sci. Adv. **9**, eadg1894 (2023)
DOI: 10.1126/sciadv.adg1894

This PDF file includes:

Figs. S1 to S21
Tables S1 to S3

Figure S1

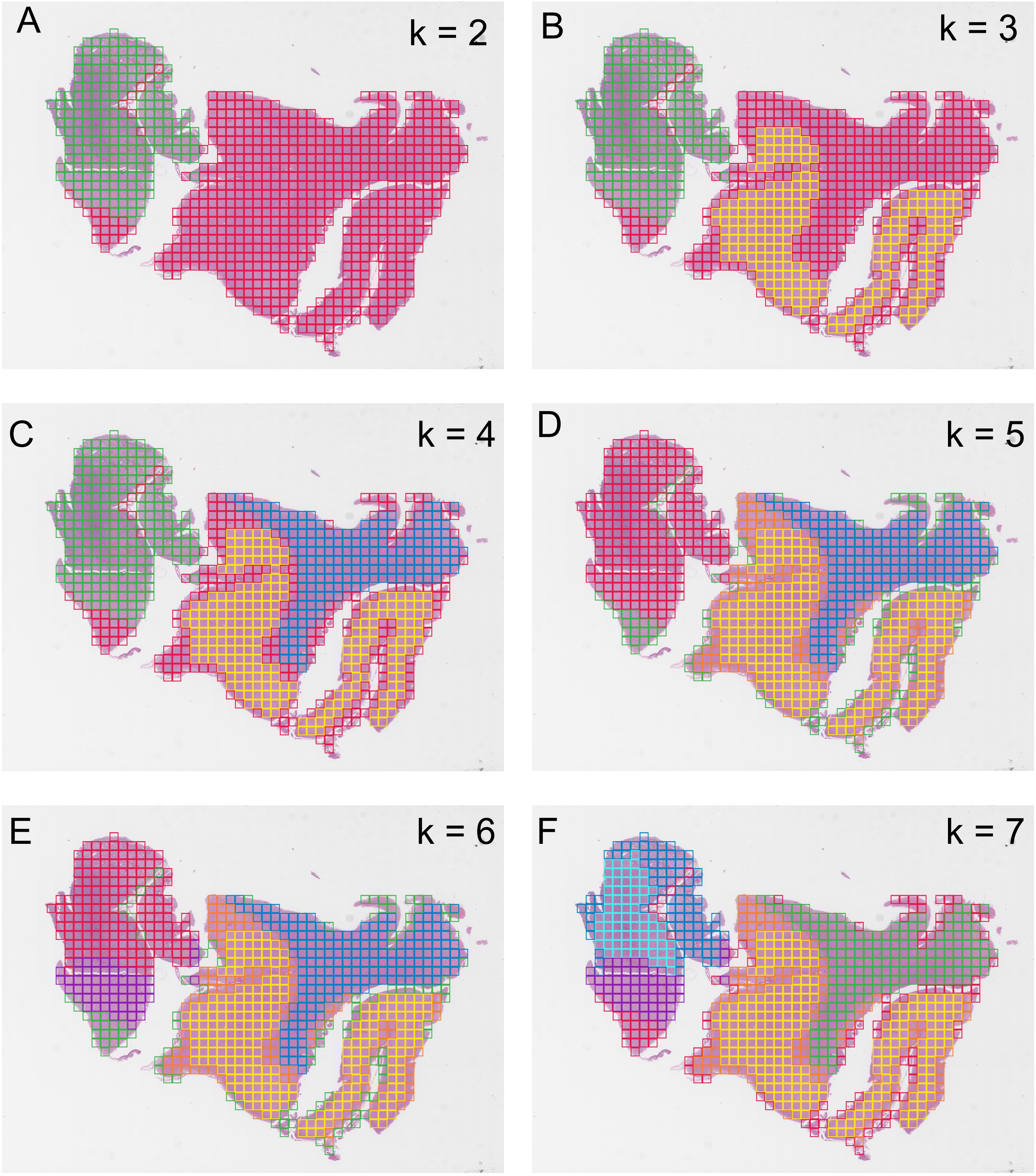

Fig. S1.

Sequential image-based clustering solutions resolve different degrees of heterogeneity across WSIs. Clustering of a diffuse glioma WSI with partition solutions ranging from k=2-7 (A-F). In most cases, early HAVOC partitions separate large histomorphologic differences such as tumoral vs normal brain tissue (green versus red, k=2). Additional partitions allow for the identification of more subtle histomorphological differences, such as glioma regions with and without edema (blue vs. cyan, k=7, respectively). Note: the unsupervised nature of this analysis results in similar regions having different color labels at different partition solutions (k values).

Figure S2

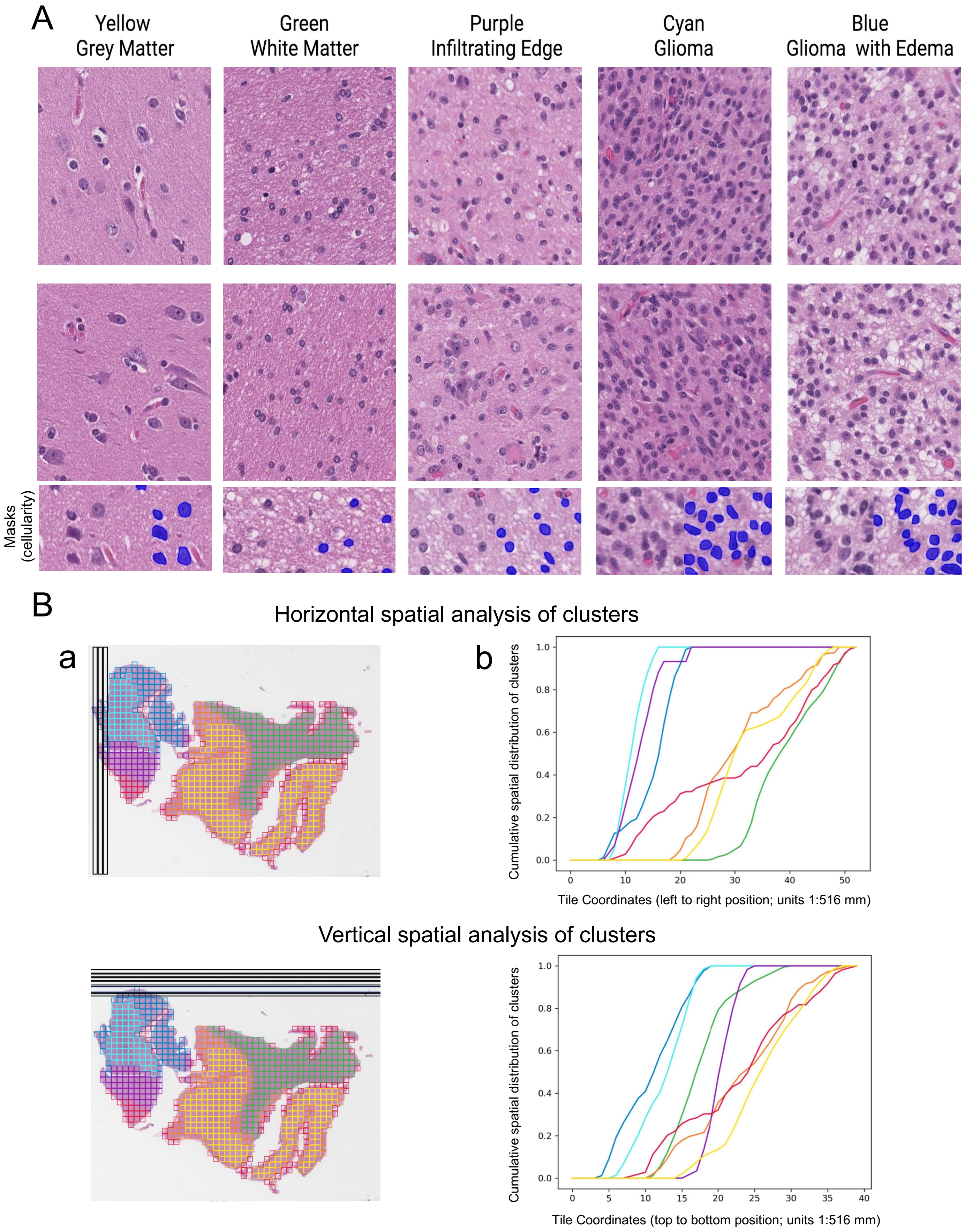
A
Yellow
Grey Matter
Green
White Matter
Purple
Infiltrating Edge
Cyan
Glioma
Blue
Glioma with Edema
Masks
(cellularity)
B
Horizontal spatial analysis of clusters
a
b
Cumulative spatial distribution of clusters
Tile Coordinates (left to right position; units 1:516 mm)
Vertical spatial analysis of clusters
Cumulative spatial distribution of clusters
Tile Coordinates (top to bottom position; units 1:516 mm)

Fig. S2.

HAVOC resolution of intra-tumoral niches in diffuse glioma. (A) Representative image patches from the discrete histomorphologic HAVOC partitions for the slide shown in Fig. 1. Regions align with human-defined glioma niches including cellular tumor, infiltrating tumor, white and gray matter. More subtle areas with tumoral edema are also detected. Dimensions of image patches shown: 0.27 mm^2; Sample masks from Mask R-CNN cellularity analysis are provided for reference (Dimensions: 4,218μm^2). (**B**) (a) Cartoons highlighting how spatial distribution differences of tumor patterns across entire slide are analyzed. In the horizontal spatial analysis, we counted the cumulative number of tiles for each color from left to right. For the vertical analysis, we countered the cumulative number of tiles for each color from top to bottom. (**b**) Horizontal and vertical cumulative distribution plot of the overall fraction of tiles from each HAVOC-defined cluster in Fig. 1E across entire WSI highlighting non-random spatial distributions of proposed histomorphologies. The Kolmogorov–Smirnov statistic of distribution of cellular $tumor_{cyan}$ vs infiltrating $tumor_{purple} = 0.375$ (p=0.0068). The Kolmogorov–Smirnov statistic of horizontal distribution of cellular $tumor_{cyan}$ vs cellular tumor with $edema_{blue} = 0.69$ (p=1.2e^{-11}). These plots and values are also in agreement with the qualitative impression that the tumor regions occupy the upper left regions of the slide (blue/cyan).

Figure S3

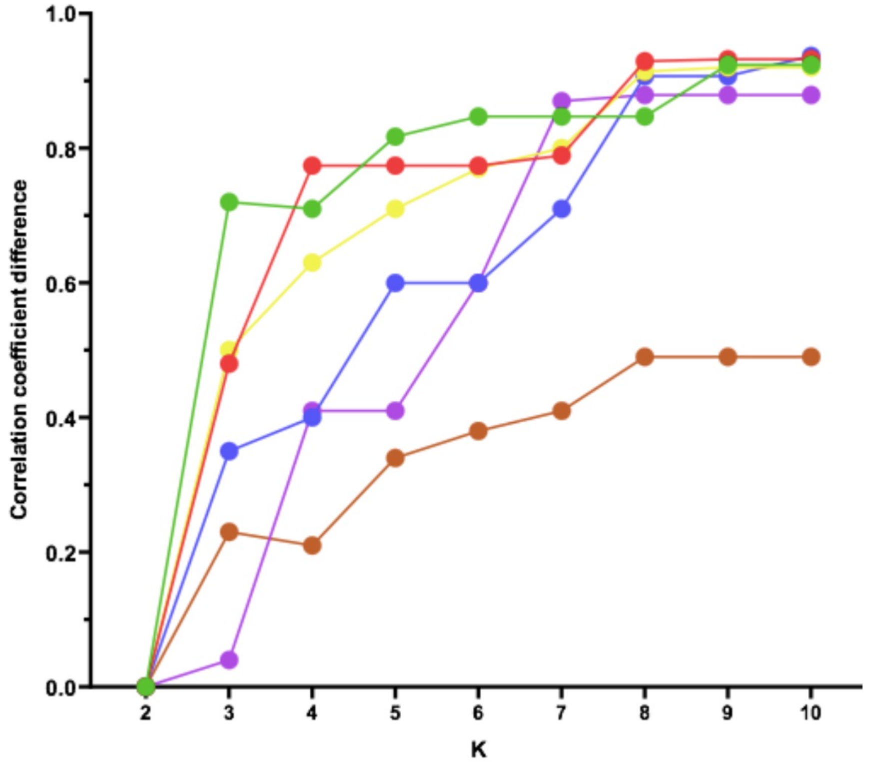

1.0
0.8
0.6
0.4
0.2
0.0
Correlation coefficient difference
2
3
4
5
6
7
8
9
10
K

Fig. S3.

Highest DLFV *r* between HAVOC-defined partitions across six representative WSI increasing from k=2 to k=10. In the majority of encountered specimen, irrespective of the initial partition DLFV *r* correlation, the degree of discernible histomorphologic heterogeneity (DLFV *r* ~0.74 that aligns with “moderate” morphologic heterogeneity) reaches saturation after ~6-8 WSI partitions. This is in agreement with the observation that early partitions usually separate out regions with large histomorphologic differences (higher DLFV r values), while later partitions define more subtle sub-patterns (tumor vs tumor with edema) with higher DLFV correlation values (See Fig. S1 and Fig. 1H for a demonstrative example). Note: Each of the six colored lines in plot highlights this Highest DLFV *r* vs k relationship in independent representative cases.

Figure S4

A

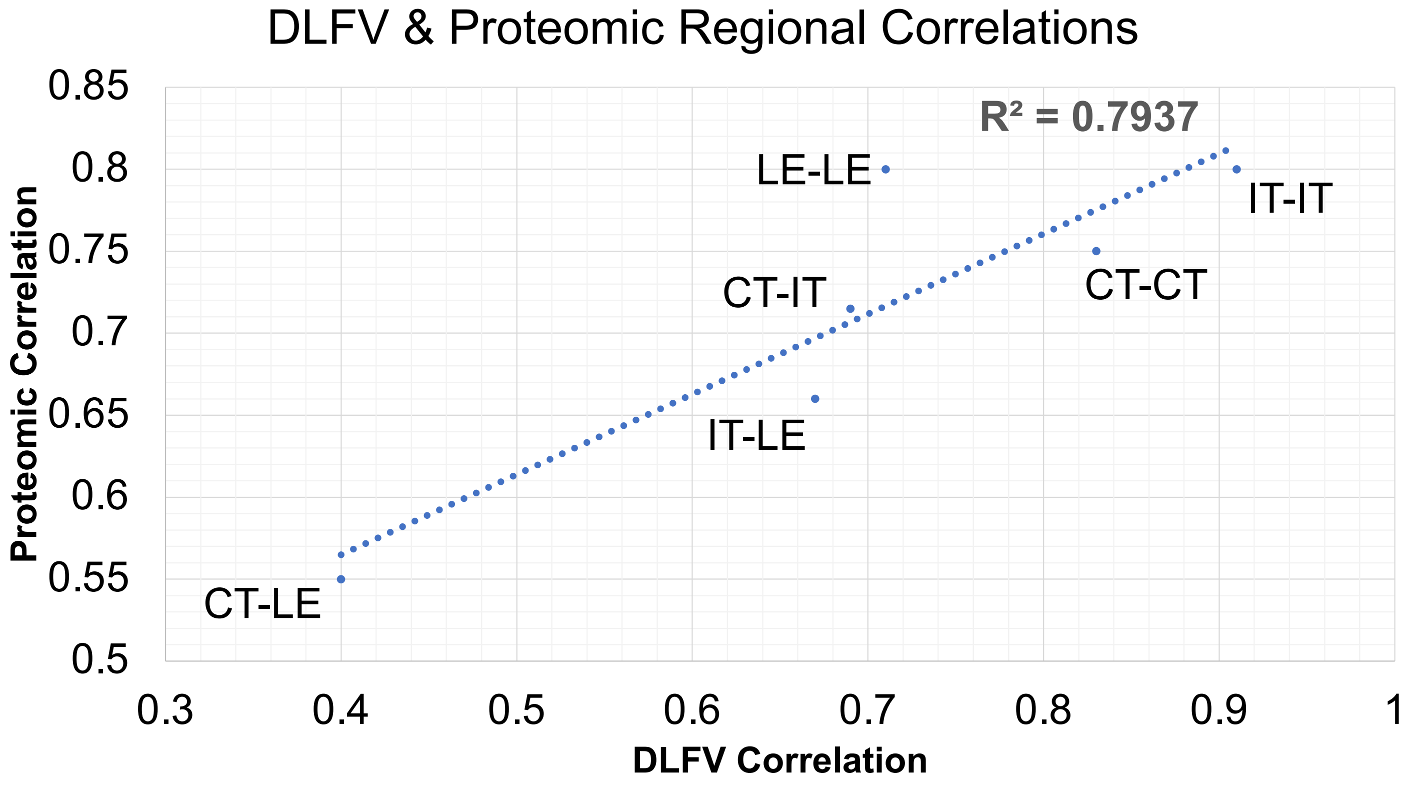
DLFV & Proteomic Regional Correlations
R² = 0.7937
LE-LE
IT-IT
CT-CT
CT-IT
IT-LE
CT-LE
Proteomic Correlation
DLFV Correlation
0.85
0.8
0.75
0.7
0.65
0.6
0.55
0.5
0.3
0.4
0.5
0.6
0.7
0.8
0.9
1

B

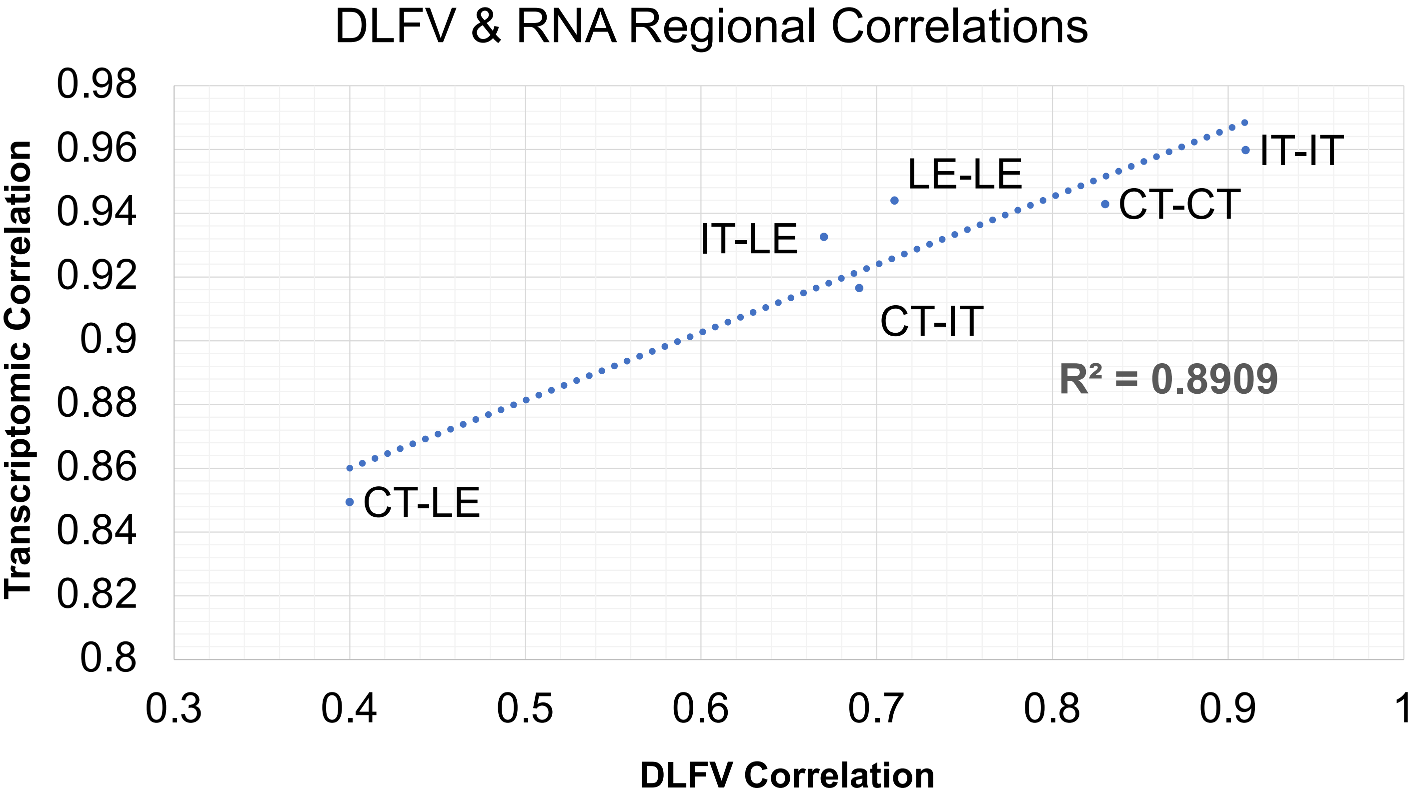
DLFV & RNA Regional Correlations
IT-IT
LE-LE
CT-CT
IT-LE
CT-IT
R² = 0.8909
CT-LE
Transcriptomic Correlation
DLFV Correlation
0.98
0.96
0.94
0.92
0.9
0.88
0.86
0.84
0.82
0.8
0.3
0.4
0.5
0.6
0.7
0.8
0.9
1

Fig. S4.

Molecular expression patterns and DLFVs covary across diffuse glioma niches. Comparison of region-to-region (A) proteomic and (B) transcriptomic versus DLFV Pearson correlations (*r*) between well-defined glioma niches (leading edge (LE), infiltrating tumor (IT), and cellular tumor (CT)). Both modalities show a strong correlation. Proteomic data retrieved from Lam et al.(*33*) and transcriptomic data taken from the Ivy Gap database (*34*).

Figure S5

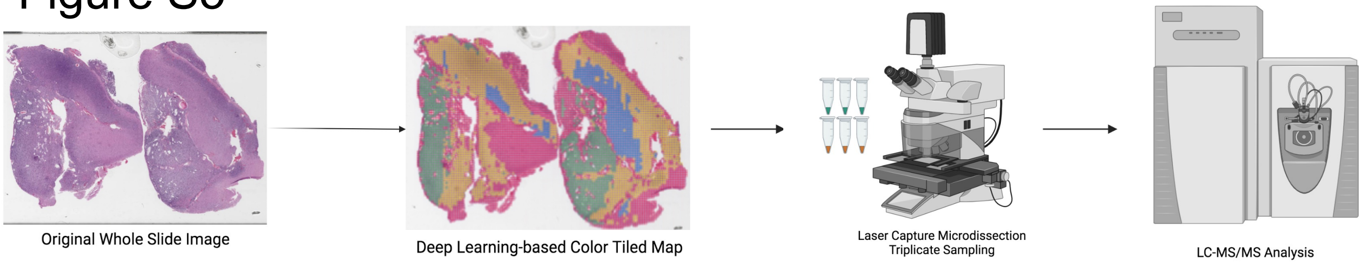
Original Whole Slide Image
Deep Learning-based Color Tiled Map
Laser Capture Microdissection
Triplicate Sampling
LC-MS/MS Analysis

Fig. S5.

Schematic of HAVOC profiling workflow. H&E-stained WSI are assessed by HAVOC to define spatial distribution of intra-tumoral histomorphological differences. Cases with large HAVOC-defined regions are subsequently isolated with laser-capture microdissection and prepared for LC-MS/MS proteomic profiling.

Figure S6

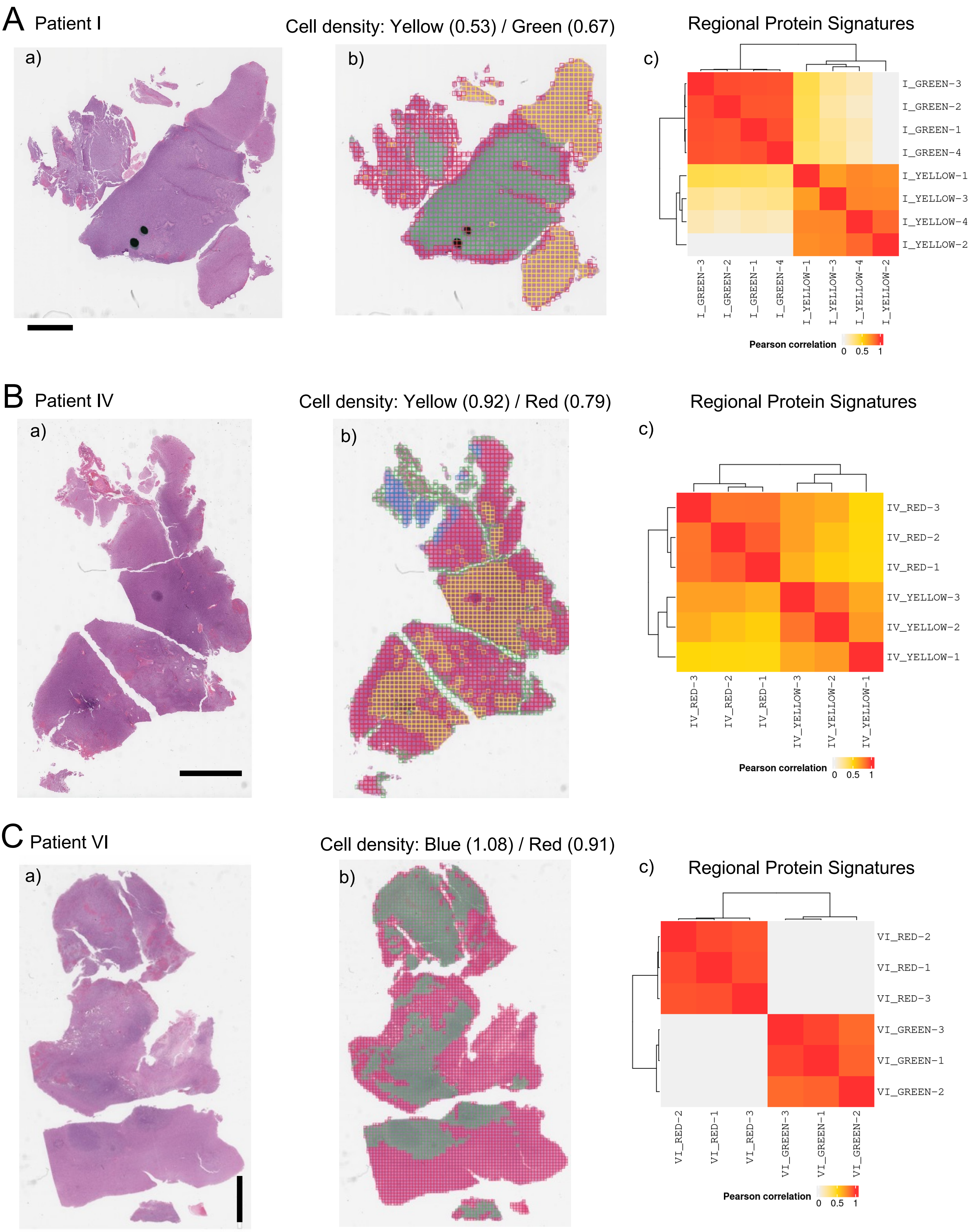

A Patient I
Cell density: Yellow (0.53) / Green (0.67)
Regional Protein Signatures
a)
b)
c)
I_GREEN−3
I_GREEN−2
I_GREEN−1
I_GREEN−4
I_YELLOW−1
I_YELLOW−3
I_YELLOW−4
I_YELLOW−2
Pearson correlation
0
0.5
1
B Patient IV
Cell density: Yellow (0.92) / Red (0.79)
Regional Protein Signatures
a)
b)
c)
IV_RED−3
IV_RED−2
IV_RED−1
IV_YELLOW−3
IV_YELLOW−2
IV_YELLOW−1
Pearson correlation
0
0.5
1
C Patient VI
Cell density: Blue (1.08) / Red (0.91)
Regional Protein Signatures
a)
b)
c)
VI_RED−2
VI_RED−1
VI_RED−3
VI_GREEN−3
VI_GREEN−1
VI_GREEN−2
Pearson correlation
0
0.5
1

Fig. S6.

Mapping of spatial protein-based signature differences in high grade glioma using HAVOC. Additional examples of proteomic spatial differences in individual cases of diffuse gliomas guided by HAVOC mapping. Each panel represent independent cases with a single slide per patient (See Table S1 for clinical information). (**A**) H&E-stained sections, (**B**) HAVOC maps and (**C**) hierarchical clustering of the LC-MS/MS proteomic correlation matrices from each region are shown for each case. In all examples, the 3-4 replicate regions were excised from independent slides and analyzed separately. These regions show consistent differences across multiple sections. The relative cellular density of each region is also provided for reference (estimated by QuPath as indicated in the methods section).

Figure S7

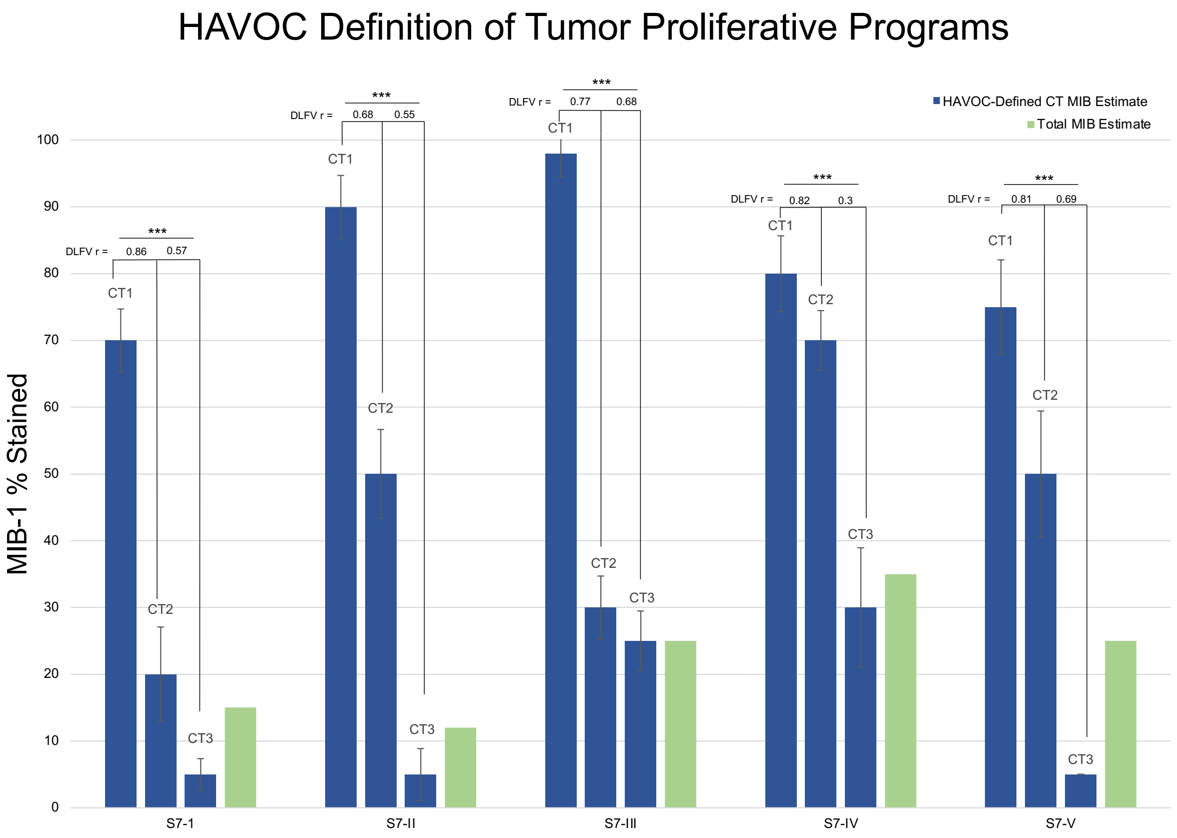
HAVOC Definition of Tumor Proliferative Programs
HAVOC-Defined CT MIB Estimate
Total MIB Estimate
MIB-1 % Stained
0
10
20
30
40
50
60
70
80
90
100
S7-1
S7-II
S7-III
S7-IV
S7-V
DLFV r =
0.86
0.57
***
CT1
CT2
CT3
DLFV r =
0.68
0.55
***
CT1
CT2
CT3
DLFV r =
0.77
0.68
***
CT1
CT2
CT3
DLFV r =
0.82
0.3
***
CT1
CT2
CT3
DLFV r =
0.81
0.69
***
CT1
CT2
CT3

Fig. S7.

Histomic heterogeneity defined by HAVOC distances (DLFV r) correlates with variable proliferation patterns in high grade gliomas. Relationship between estimated proliferation indices (Ki-67) and regional DLFV-generated *r* values across HAVOC cellular tumor-enriched partitions (CT1, CT2, etc…)(n=5, *** denotes p<0.0001). Each blue bar represents a different HAVOC partition from high grade glioma cases that show spatially variable proliferation rates. For reference, an overall Ki-67 proliferation index over the entire tissue slide is also provided (green bar). Overall, this later metric underestimates the proliferative potential of many spatial tumor sub-compartments. Note: Case numbers (SF-I, SF-II,…) correspond to patient IDs shown in Table S2. DLFV *r* values between different HAVOC partitions (cellular tumor regions only) are provided above the bars and are in relation to CT1.

Figure S8

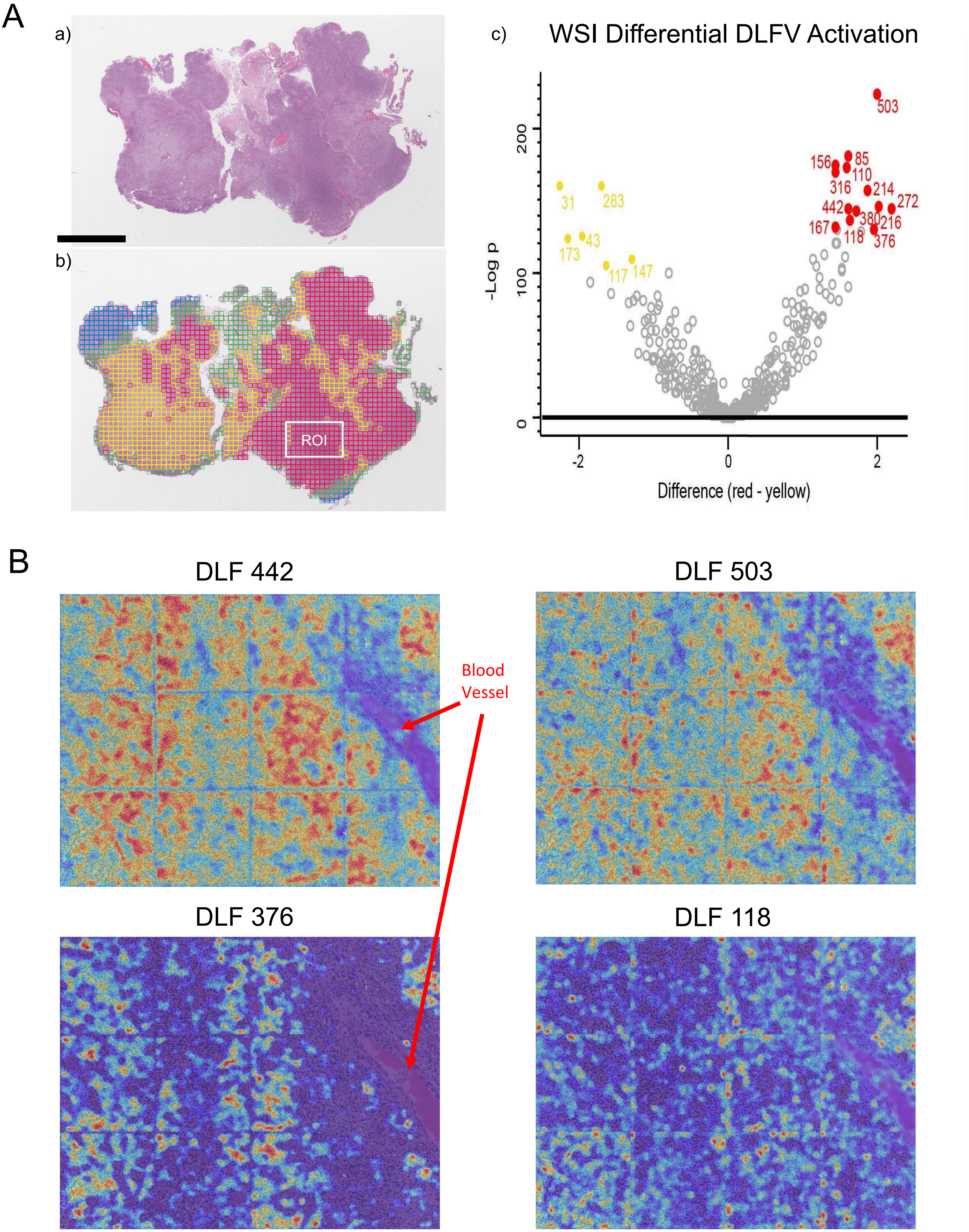
A
a)
b)
ROI
c)
WSI Differential DLFV Activation
-Log p
Difference (red - yellow)
0
100
200
-2
0
2
503
156
85
110
316
214
272
442
380
216
167
118
376
31
283
173
43
117
147
B
DLF 442
DLF 503
Blood Vessel
DLF 376
DLF 118

Fig. S8.

Regional variation of deep learning features in a glioblastoma. (A) Volcano plot (subpanel c) highlighting differential DLF activation between the HAVOC-defined partitions. The H&E (subpanel a) and HAVOC map (subpanel b) from Fig. 2 are included for reference. (**B)**. Representative feature activation maps of DLFs enriched in the RED region (ROI from panel **A**(b). Some DLFs show diffuse activation patterns across the entire tumor (DLF 442/503) while other DLFs show a more punctate pattern of signal (DLF376, DLF118). As these DLFs represent features asymmetrically enriched in neighboring tumor regions, it is expected that they will often represent complex tissue/cellular patterns and give a relatively non-uniform activation pattern. Moreover, while some of these patterns show overlapping spatial coordinates, individual DLFs appear to capture different salient regions of the sample tumor area. Note: all DLFs shown are fairly specific for tumor regions and are not activated in areas with non-neoplastic elements (e.g. blood vessels).

Figure S9

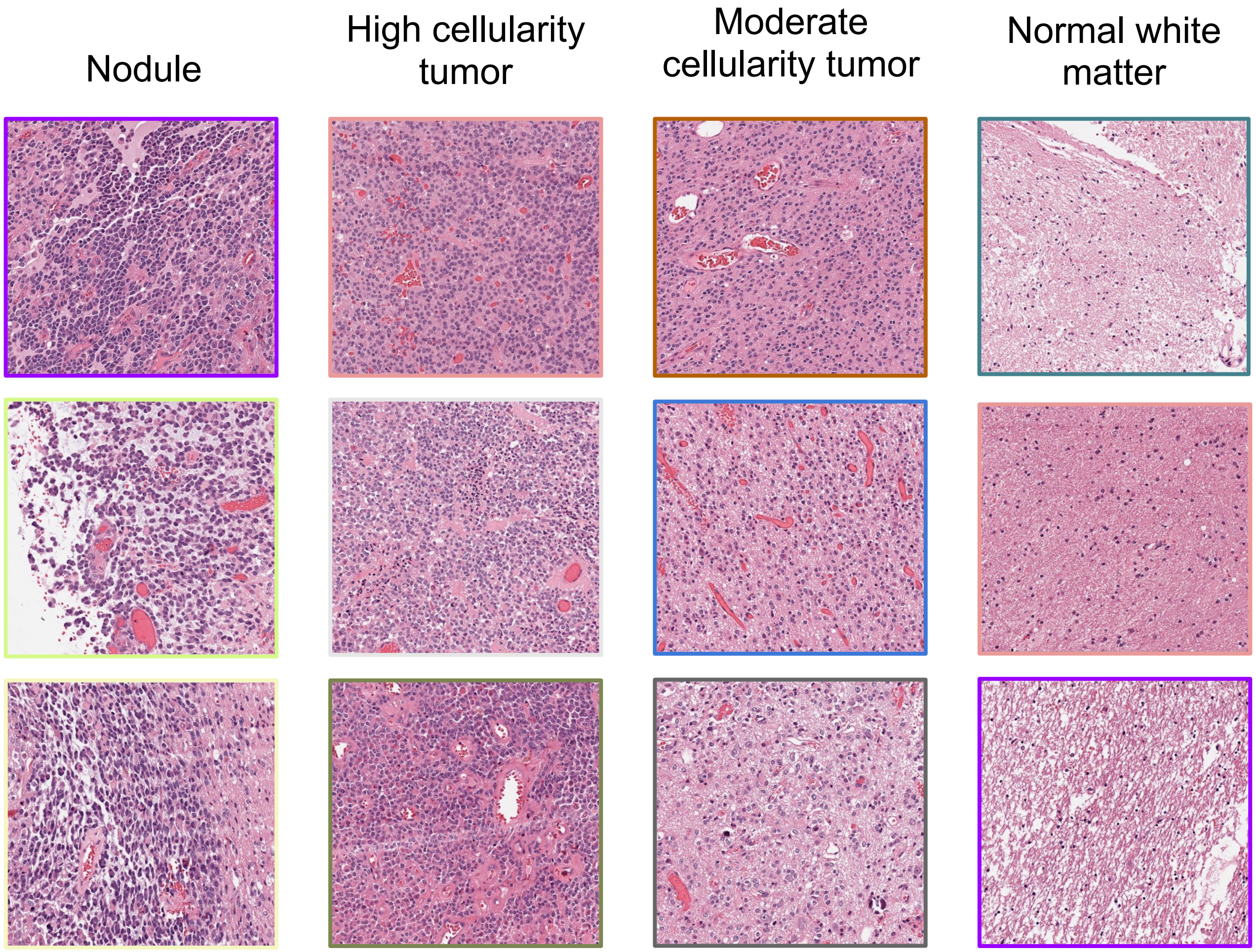
Nodule
High cellularity tumor
Moderate cellularity tumor
Normal white matter

Fig. S9.

Representative H&E images patches from different clustered regions from Fig. 3. Image patch border colors correspond to their originating WSI color assigned in Fig. 3A. Dimensions of image patches shown: 0.27 mm^2.

Figure S10

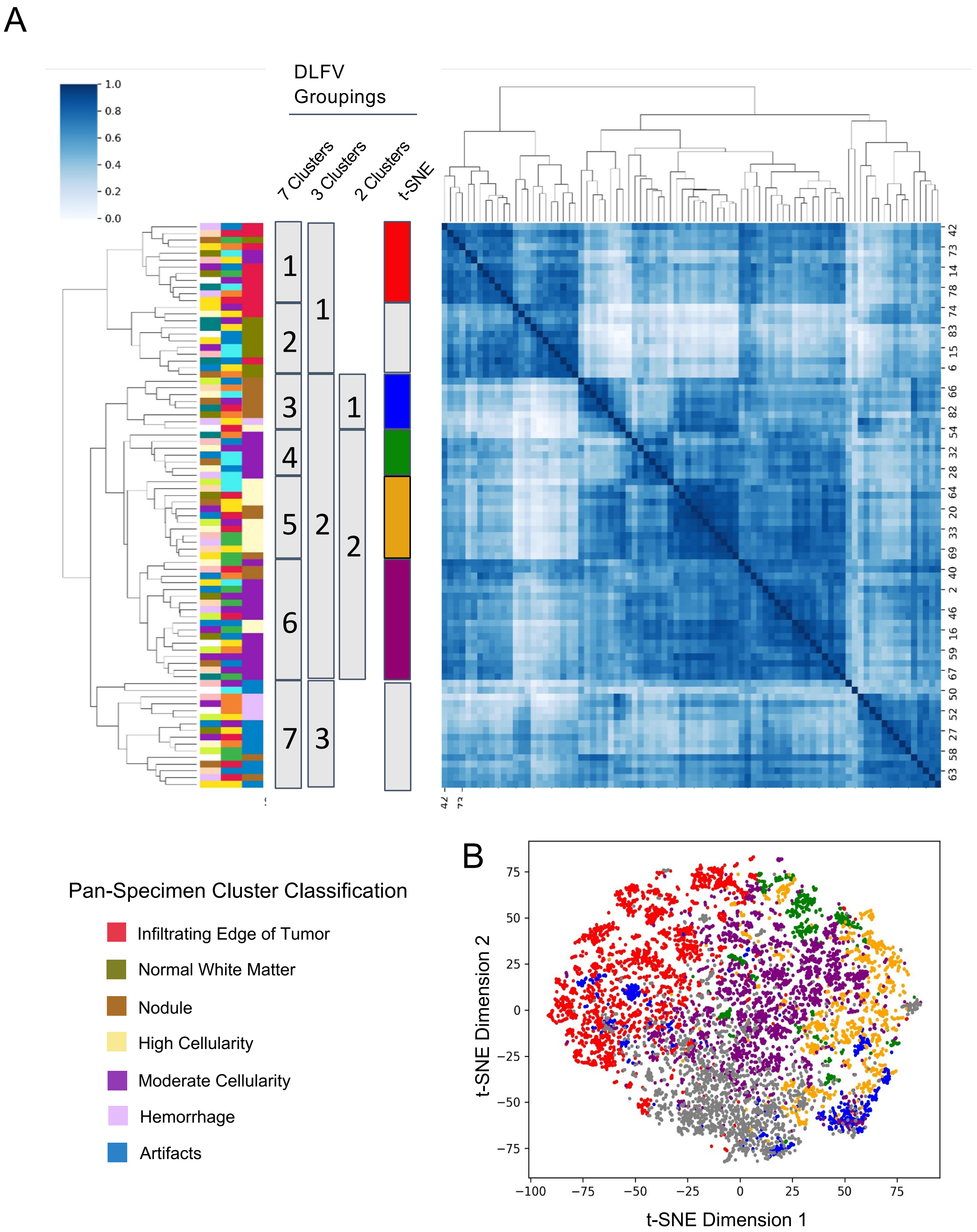

Note: Colours correspond to cluster IDs shown in Panel A

Fig. S10.

DLFV-based groupings closely align with human annotations. (A) Pairwise Pearson correlation matrix arranging all 84 clusters from **Fig. 3**. This version displays consensus clusters derived by inspection of the hierarchical clustering tree combined with chi-squared goodness of fit testing to define solutions with clusters disproportionately skewed from the random distribution. In the 7-cluster solution, there is significant enrichment for infiltrating edge of tumor (cluster 1), normal white matter (cluster 2), tumor regions with nodularity (cluster 3), moderately cellular tumor (clusters 4&6), higher cellular tumor regions (cluster 5) and artifacts/tissue edges/hemorrhage (cluster 7). In the 3-cluster solution, regions were skewed from random with largely non-neoplastic/inter-mixed brain tissue regions grouping together (cluster 1), cellular neoplastic regions forming a large group (cluster 2) and a third grouping largely comprised of tissue artifacts and hemorrhage (cluster 3). Focusing only on the tumor-enrich cluster, there was a significant division in regions showing higher cellularity with nodularity (cluster 1) and a large tumor region largely comprised of non-nodular tumor regions (cluster 2). The statistical testing for all these solutions can be found in table S3. We also provide a color scheme used to visualize the positional organization of tiles within a t-SNE plot shown in panel **B**. (**B**) t-SNE plot of all 10,973 0.27 mm^2 image patches from the entire tumor in **Fig. 3**. Coloured data points correspond to image patches belonging to HAVOC partitions shown in panel **A**. From right to left, there is a gradient transitioning from patches derived from the cluster enriched in hyper-cellular nodules (blue cluster), the cluster enriched in cellular tumor (orange), clusters enriched with moderate cellular tumor regions (purple/green) and the tissue regions enriched for low tumor cellularity (red). Gray points highlight non-cellular/tumor clusters (hemorrhage/edge tiles). Note: This color scheme is different from that found in **Fig. 3** as these colors are meant to represent patches corresponding to cluster IDs rather than the specific human histological annotations (different color scheme).

Figure S11

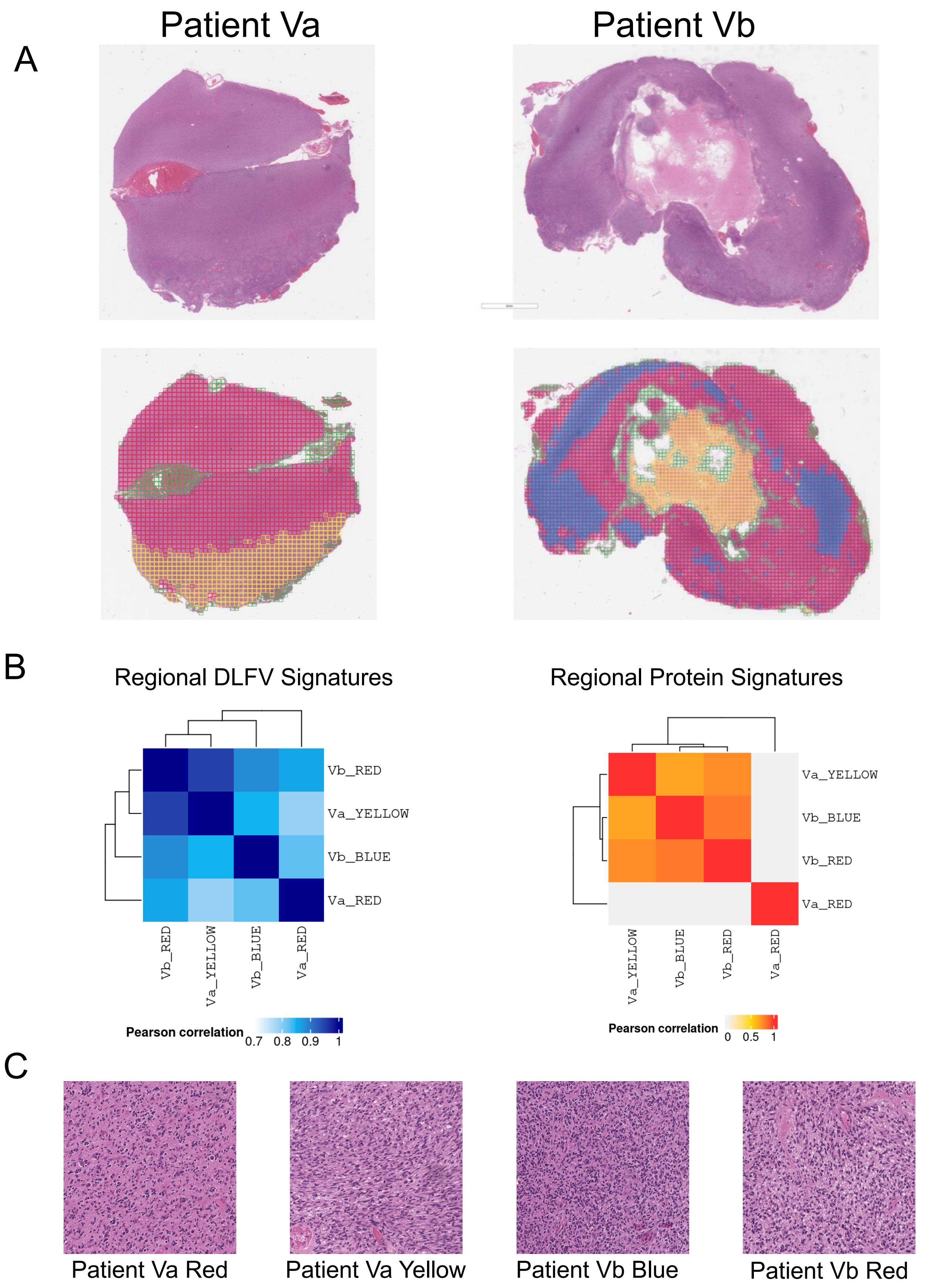
Patient Va
Patient Vb
A
B
Regional DLFV Signatures
Regional Protein Signatures
Vb_RED
Va_YELLOW
Vb_BLUE
Va_RED
Pearson correlation
0.7 0.8 0.9 1
Va_YELLOW
Vb_BLUE
Vb_RED
Va_RED
Pearson correlation
0 0.5 1
C
Patient Va Red
Patient Va Yellow
Patient Vb Blue
Patient Vb Red

Fig. S11

HAVOC mapping across independent slides align with overall molecular correlations (A) Another example of a slide pair analyzed concurrently by HAVOC and molecular analysis. **(B)** Integrated DLFV correlation matrices across both slides define a distinct infiltrative compartment (Va_RED) and a more cellular tumor region on initial slide (Va_YELLOW). The accompanying slide shows 2 proposed tumor patterns encircling a central necrotic region. These multi-slide maps are in agreement with global patterns of proteomic variations derived from each of the major HAVOC defined partitions. Overall, there is a distinct infiltrative compartment and three fairly similar regions of high tumor cellularity. The closer pairing of Va_RED and Vb_YELLOW in the DLFV r map is in agreement with the cellular density quantifications shown in Fig. S12. (**C**) Representative image patches from these HAVOC partitions provided to highlight qualitative morphological differences are in agreement with the relative HAVOC and molecular differences.

Figure S12

Patient II

Group	Region (avg. cell density and SD)	
	IIa	IIb
Ultra-high	-	BLUE (1.31±0.09)
High	GREEN (0.83±0.07)	RED (0.94±0.04)
Mid	YELLOW (0.67±0.08)	-
Low	-	YELLOW (0.44±0.06)

Block IIa

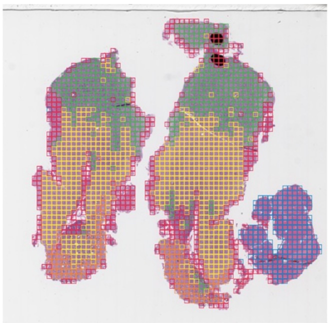

Block IIb

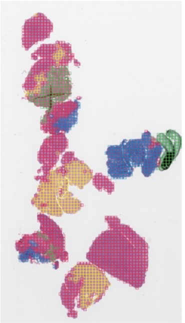

Patient III

Group	Region (avg. cell density and SD)	
	IIIa	IIIb
Ultra-high	-	-
High	-	-
Mid	YELLOW (0.60±0.05)	GREEN (0.72±0.16)
Low	GREEN (0.46±0.07)	RED (0.51±0.09)

Block IIIa

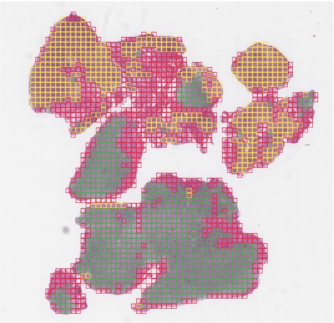

Block IIIb

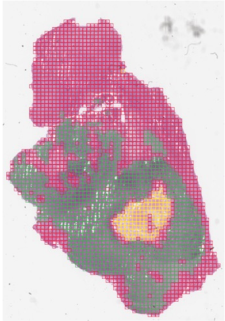

Patient V

Group	Region (avg. cell density and SD)	
	Va	Vb
Ultra-high	-	-
High	YELLOW (0.85±0.11)<br>RED (0.79±0.12)	BLUE (1.09±0.10)<br>RED (0.91±0.14)
Mid	-	-
Low	-	-

Block Va

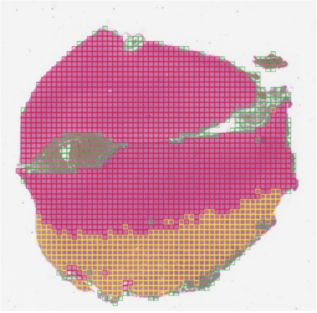

Block Vb

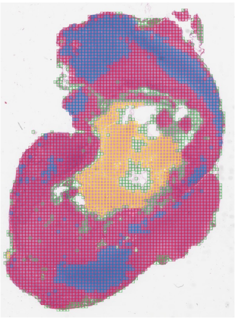

Fig. S12.
Regional cell density estimates align with HAVOC mapping across independent slides. Similarities of cell densities of closely clustered regions also supports the non-random partitioning and grouping of regions by HAVOC. Numbers represent estimates using QuPath, as indicated in the methods section.

Figure S13

64-signature gene expression analysis

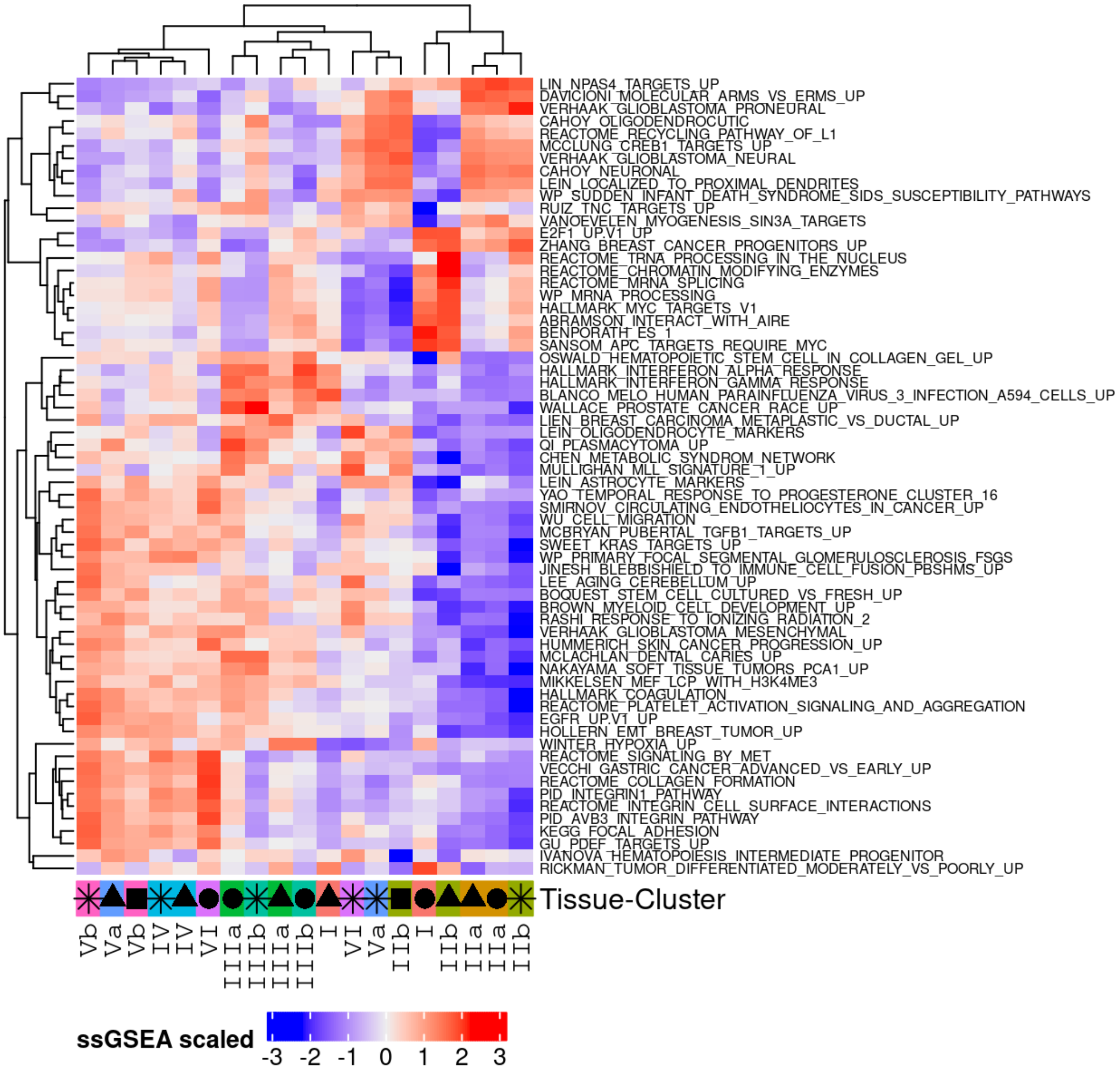

Fig. S13.

Hierarchical clustering of proteomics signatures. Enrichment of 64 pre-selected genesets that were previously deemed informative in GBM (See Ref (*33*)) was calculated by ssGSEA and followed by hierarchical clustering. This unsupervised approach results in samples being mainly grouped by tissue, and with limited intra-sample regional separation. Overall, there are intra-tumoral regional biological pathway differences identified in practically all patients within the assembled cohort.

Figure S14

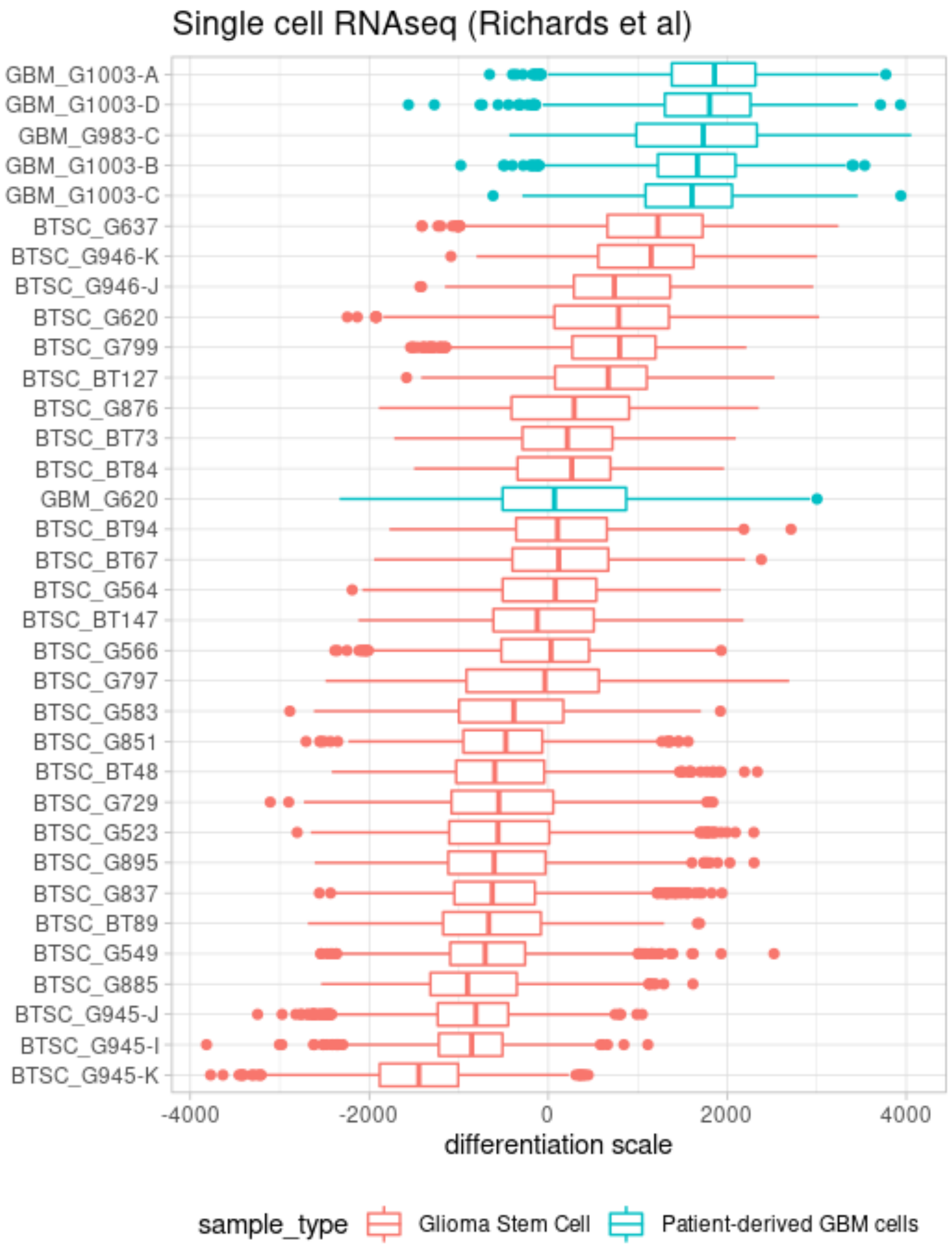
Single cell RNAseq (Richards et al)
GBM_G1003-A
GBM_G1003-D
GBM_G983-C
GBM_G1003-B
GBM_G1003-C
BTSC_G637
BTSC_G946-K
BTSC_G946-J
BTSC_G620
BTSC_G799
BTSC_BT127
BTSC_G876
BTSC_BT73
BTSC_BT84
GBM_G620
BTSC_BT94
BTSC_BT67
BTSC_G564
BTSC_BT147
BTSC_G566
BTSC_G797
BTSC_G583
BTSC_G851
BTSC_BT48
BTSC_G729
BTSC_G523
BTSC_G895
BTSC_G837
BTSC_BT89
BTSC_G549
BTSC_G885
BTSC_G945-J
BTSC_G945-I
BTSC_G945-K
-4000
-2000
0
2000
4000
differentiation scale
sample_type
Glioma Stem Cell
Patient-derived GBM cells

Fig. S14.

Varying distributions along the Astro-ES axis at the single-cell level in glioma stem cells (n=28) and patient-derived GBM cells (n=6); dataset previously published by Richards et al.(*41*)

Figure S15

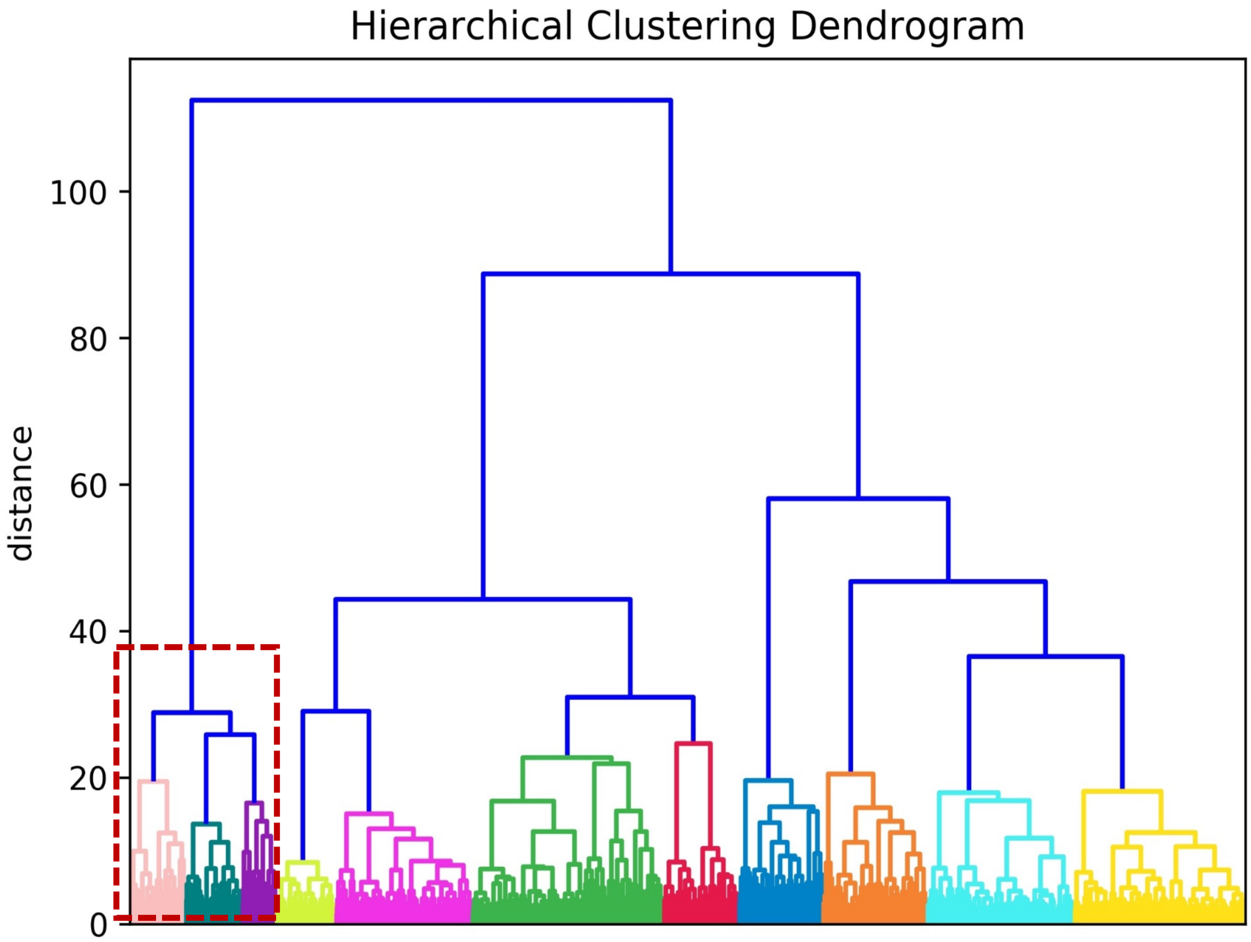
Hierarchical Clustering Dendrogram
distance
0
20
40
60
80
100

Fig. S15.

HAVOC partitioning defines metastatic subclones and peritumoral changes in an experimental lung cancer model. Hierarchical dendrogram showing the arrangement and similarities of HAVOC partitions show in Fig. 6C. While the original authors of the study could only resolve the ground truth of 2 of the five tumors tumor foci present in this model (due to throughput limitations of DNAseq), we propose a specific grouping of the remaining clones based on morphology. We note all 5 tumor foci show fairly homogenous/stable (monoclonal) pattern grouping and that the 2 molecular resolved foci are indeed separated in the dendrogram and activated by different tumor-activating (nuclear) DLFs (Fig. 6E).

Figure S16

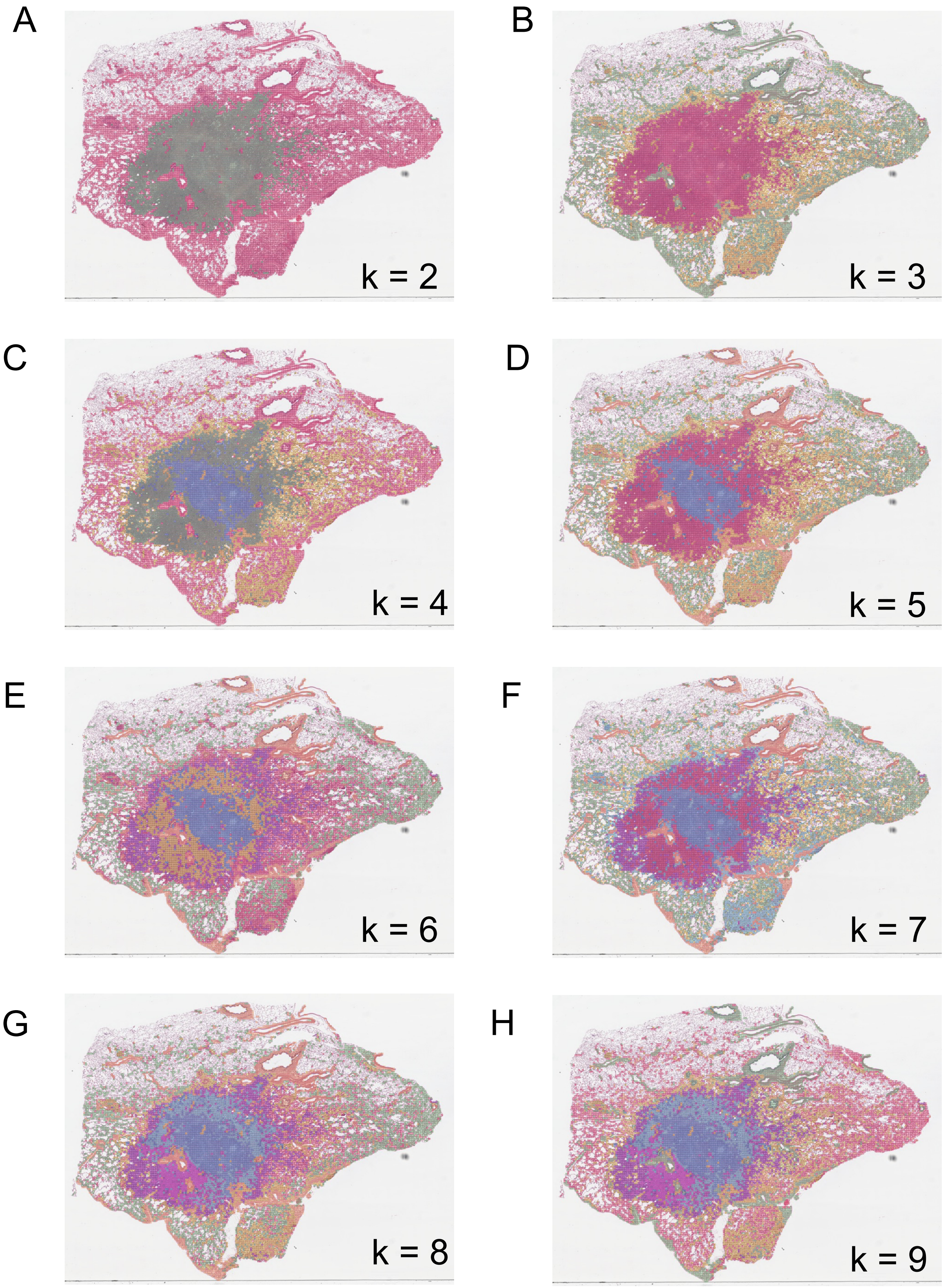

Fig. S16.

Sequential image-based clustering solutions resolve different degrees of heterogeneity across WSIs. Clustering of image patches of a WSI of a lung adenosquamous carcinoma with partition solutions ranging from k=2-9. The major squamoid pattern of morphologic heterogeneity was resolved at k=4 (blue vs green, r = 0.86). Subsequent partitions of the adenosquamous regions showed meaningful peri-tumoral HAVOC partitions around the area of squamous differentiation but these were felt to be more minor histomic differences that were not readily/objectively appreciated (Blue vs Cyan, r = 0.95, k=9).

Figure S17

A

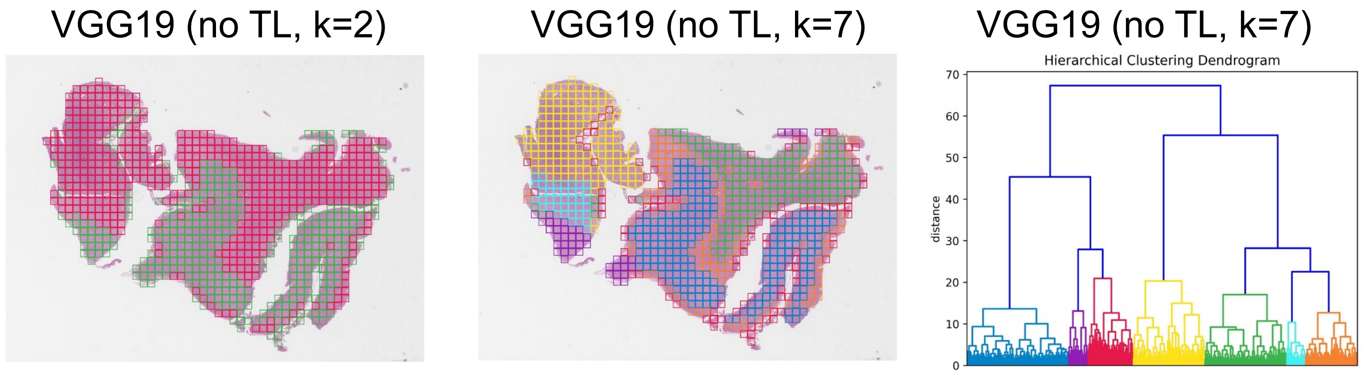

B

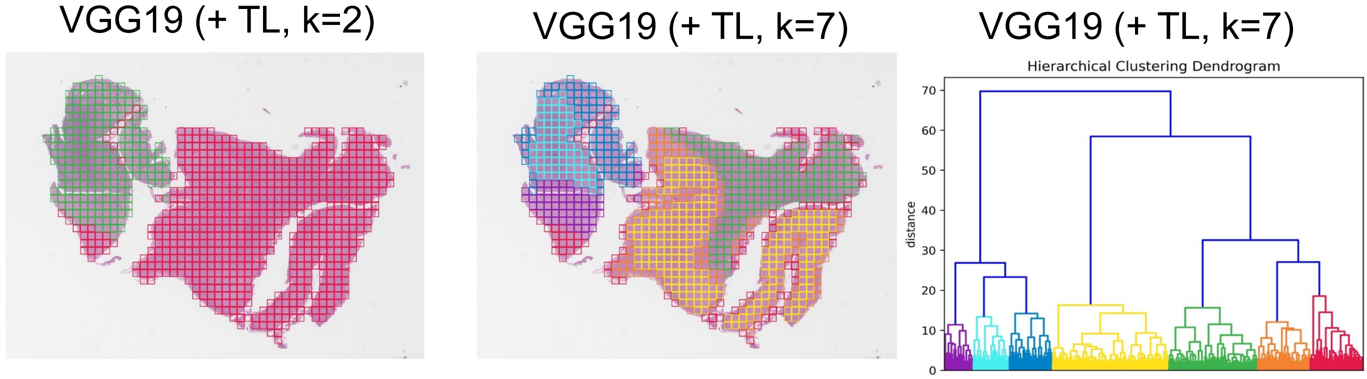

C

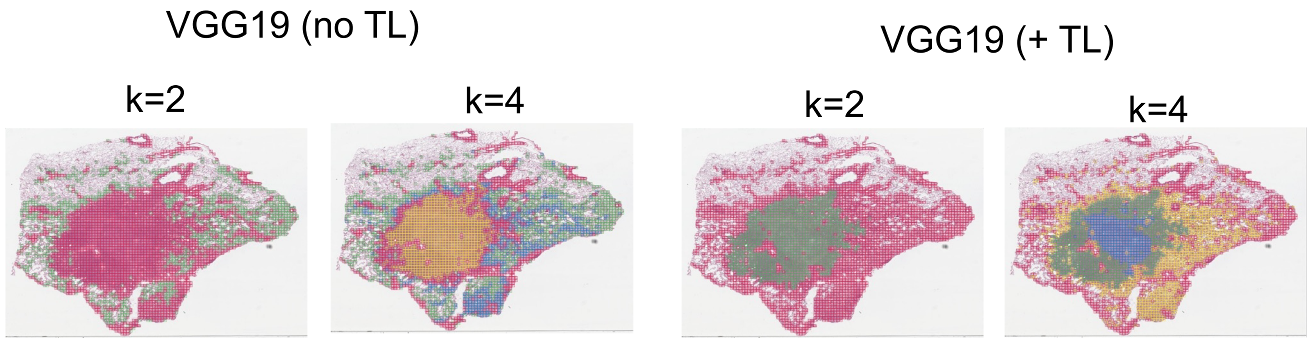

Fig. S17.

Differences in HAVOC partitions derived from CNN with and without pathology-based transfer learning. (A-B) Sample image-based clustering solutions resolved using the VGG19 CNN without and with pathology-related transfer learning (no TL / + TL). The “out-of-the-box” VGG19 showed different clustering solutions with inter-mixed neoplastic and non-neoplastic regions (k=2) and needed more than 7 total partitions to define the tumor area showing prominent edema in this slide (See Fig. 1E, and figs. S1-2 for reference). **(B)** The pathology-optimized CNN performed more intuitively separating out normal and neoplastic tissue elements and additional tumor sub-populations in early subdivisions. (**C**) These observations also held in non-brain neoplasms with more specific separation of neoplastic (TTF1+, P40+) from non-neoplastic lung tissue (TTF1-, P40-) (k=2) and the additional critical ability to detect subregions of squamous differentiation (p40+) when the tissue was partitioned using the CNN with transfer learning (blue region in k=4). Please see TTF1 and p40 immunostaining in Fig. 7C for ground truth. Dimensions of image patches shown: 0.27 mm^2.

Figure S18

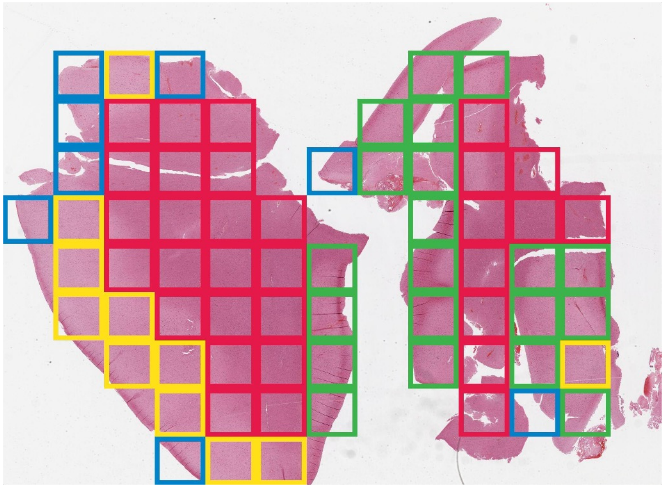

Tile Size: 4096 pixels; k = 4
Red: White matter / Yellow: Gray matter
Blue: Edge of gray matter / Green: Gray matter

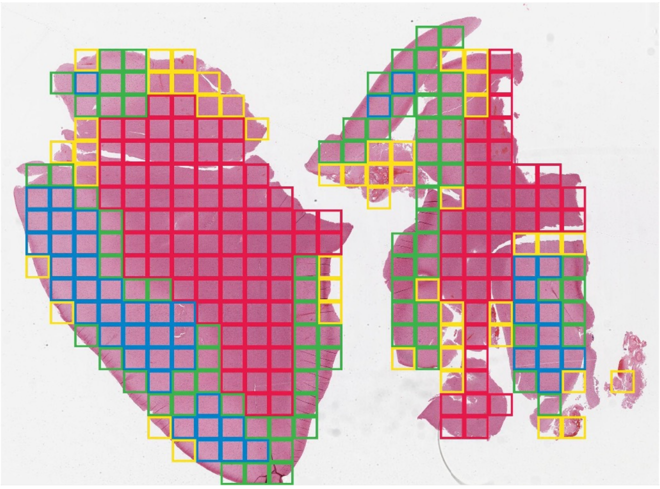

Tile Size: 2048 pixels; k = 4
Red: White matter / Yellow: Edge of gray matter
Blue: Gray matter / Green: Gray matter

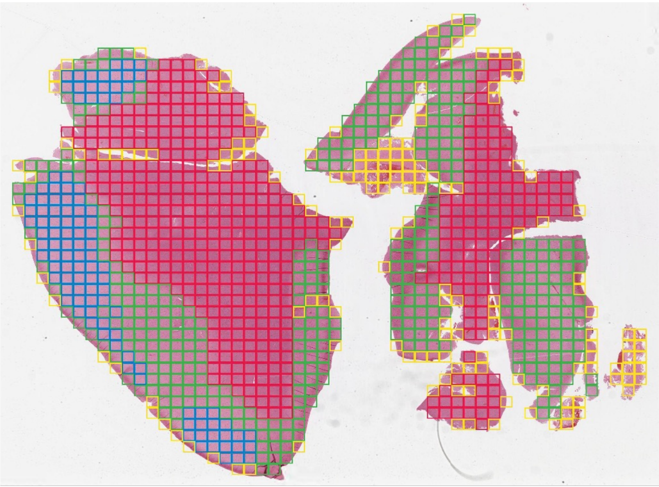

Tile Size: 1024 pixels; k = 4
Red: White matter / Yellow: Hemorrhage / Edge
Blue: Gray matter / Green: Gray matter

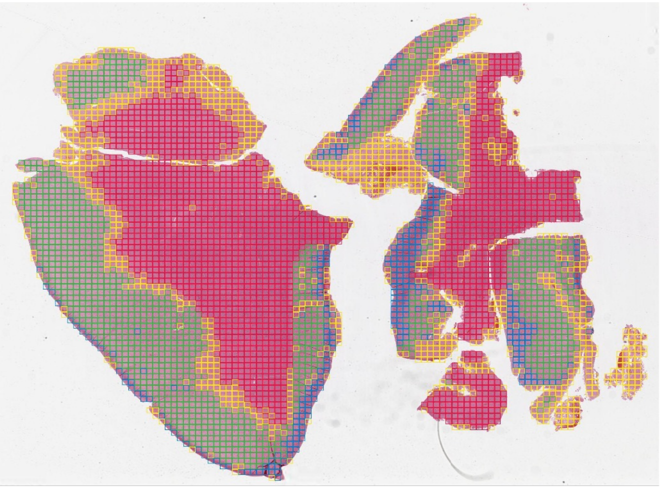

Tile Size: 512 pixels; k = 4
Red: White matter / Yellow: Gray-white matter transition
Blue: Molecular layer and darker staining gray matter
Green: Gray matter

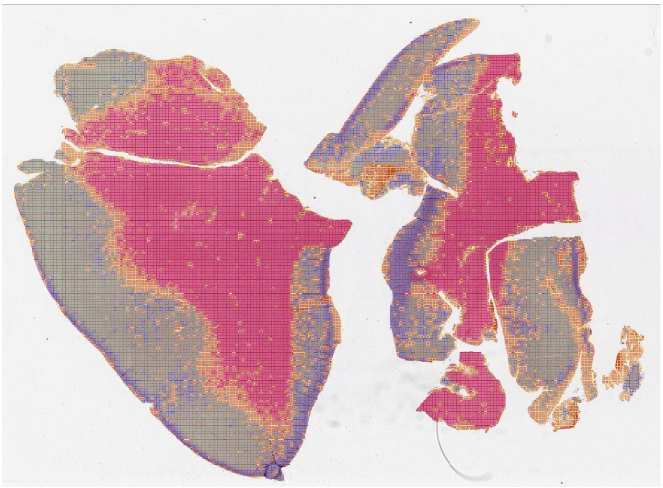

Tile Size: 256 pixels; k = 4
Red: White matter / Yellow: Gray-white matter transition
Blue: Molecular layer and darker staining gray matter
Green: Gray matter (largely layers II-VI)

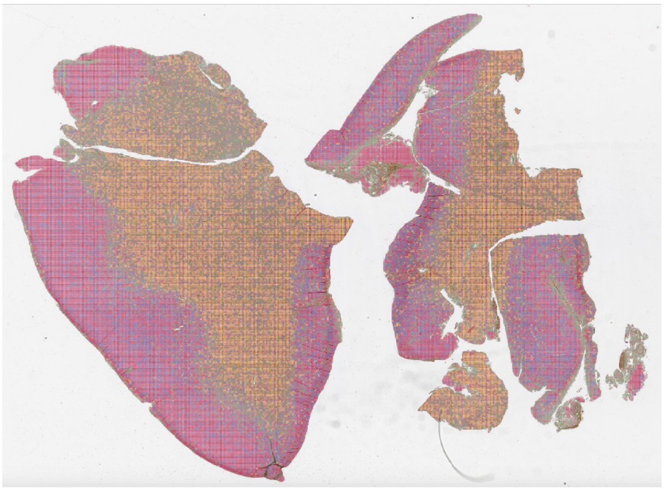

Tile Size: 128 pixels; k = 4
Red/Blue (Intermixed): Gray matter regions
Yellow/Green (Intermixed) white matter regions

Fig. S18.

Smaller tile/patch sizes improves resolution of biovariation, introduce noise and increases computational demands of HAVOC. HAVOC maps for a representative WSI containing gray and white matter (from an epilepsy case) across various tile sizes (4096, 2048, 1024, 256, 128 pixels tile width, 1 pixel = 0.505 μm) (k was set to 4 for simplicity). Human interpretations for each tile size are provided for reference. While the major tissue regions are captured at a relatively acceptable manner across almost all scales of analysis, there is an appreciable trade-off of resolution at higher tile sizes with increased noise with smaller patches (most notable at 128 pixel width). Across most cases we have attempted, this trade-off is maximized with patches with pixel widths of 256-1024; the field of view of most modern diagnostic microscopes. Notably, there is also an exponential increase in computational demand of the presented workflow with decreasing tile size having significantly longer run times and greater memory requirements.

Figure S19

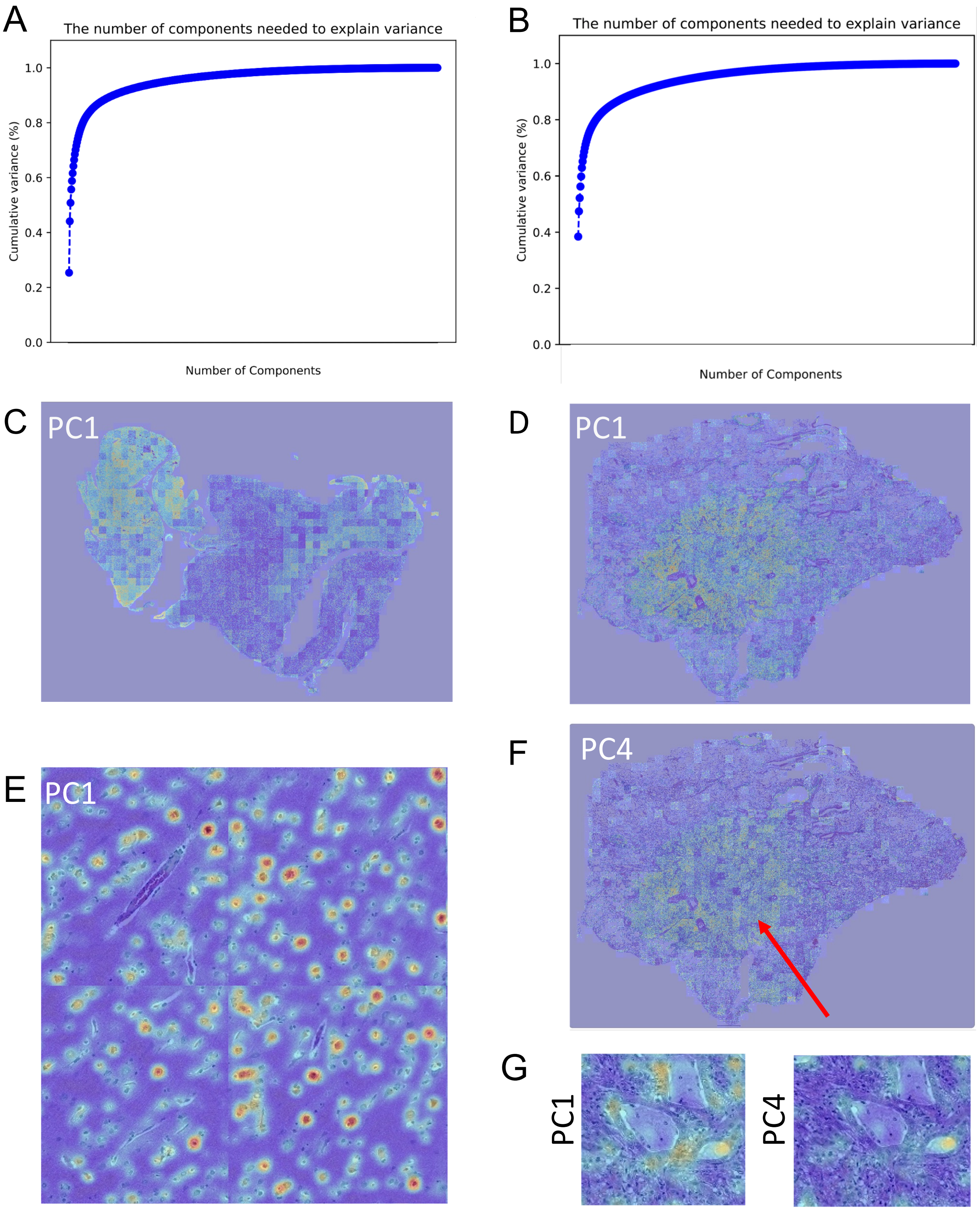
A
The number of components needed to explain variance
Cumulative variance (%)
1.0
0.8
0.6
0.4
0.2
0.0
Number of Components
B
The number of components needed to explain variance
Cumulative variance (%)
1.0
0.8
0.6
0.4
0.2
0.0
Number of Components
C
PC1
D
PC1
E
PC1
F
PC4
G
PC1
PC4

Fig. S19.

Most of the variation in deep learning feature vectors can be explained by cell-related features. (A-B) Representative cumulative variance explained as a function of principal components (PCs). Panel **A** shows cumulative variance of DLFs of the diffuse glioma presented in Fig. 1 (panels **C-I**) and panel **B** represents the adenosquamous lung carcinoma presented in Fig. 7 (panels **C-D**). 21 and 25 PCs are needed to explain 80% of the variance of WSI respectively. (**C-D)** Feature activation mapping of PC1 for respective slides highlights higher activation of the more cellular tumor regions on both WSIs. (E) Higher power view of PC1 feature activation from the diffuse glioma case shows homogenous labeling of practically all cells; with larger neuronal cell elements having higher activation (red and yellow) compared to the smaller oligodendrocyte and astrocytic cells. The activation of multiple PCs over the cellular elements suggests that much of the variation across image patches is driven by cell-related features. (**F**) PC4 feature activation of the adenocarcinoma show a relative dimming of the PC4 feature activation in the central p40+ squamous component of this tumor (arrow) compared to the peripheral TTF1+ adenocarcinoma tumor regions. This suggests to us that more complex features may be attributing to differential activation in this PC. (**G**) High power feature activation of both PC1 and PC4 in the region displaying squamous differentiation shows a reduced activation of the epithelial component of this region in PC4. Note: Feature activation of individual correlated DLFs may have more variable (punctate vs. diffuse) activation and focus on different components of the tissue section (Please see Fig. S8 and Reference 32).

Figure S20

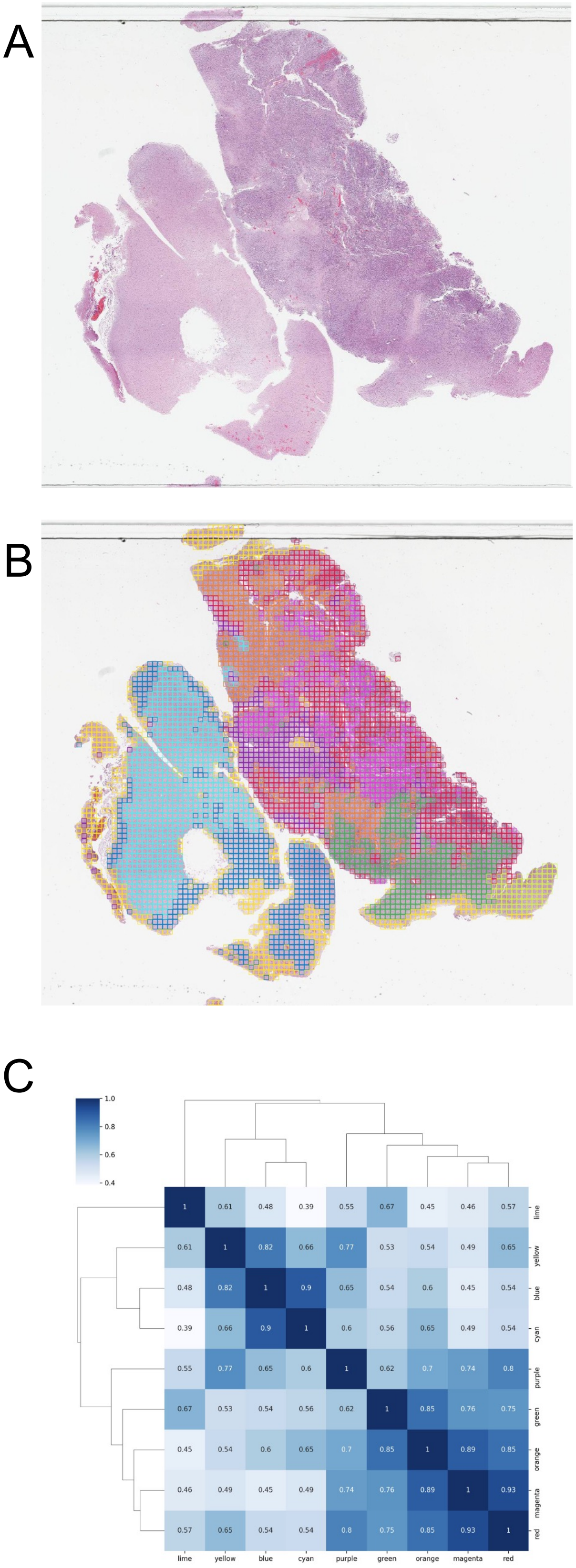

D

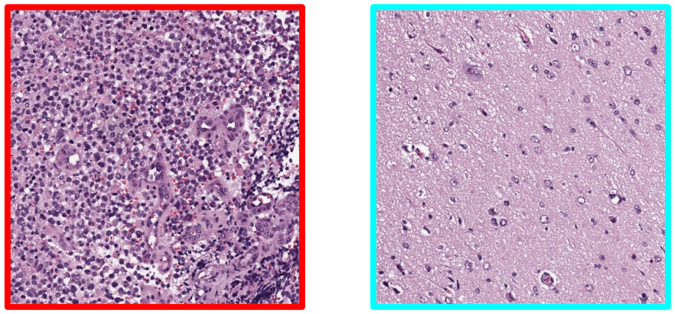

Expert Interpretation: Tumor (RED) vs Gray Matter (CYAN)
DLFV r (RED vs. CYAN): 0.54

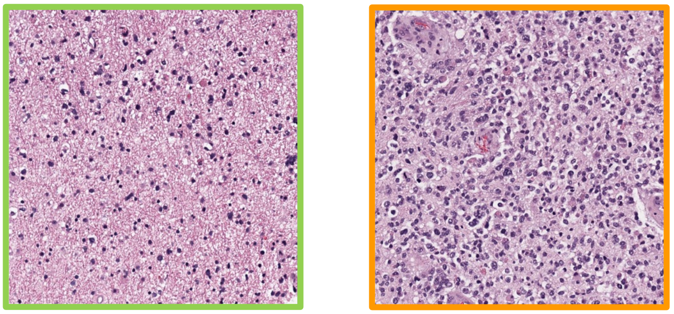

Expert Interpretation: Tumor (LIME) vs. Tumor (ORANGE) - Severe Heterogeneity
DLFV r (LIME vs. ORANGE): 0.45

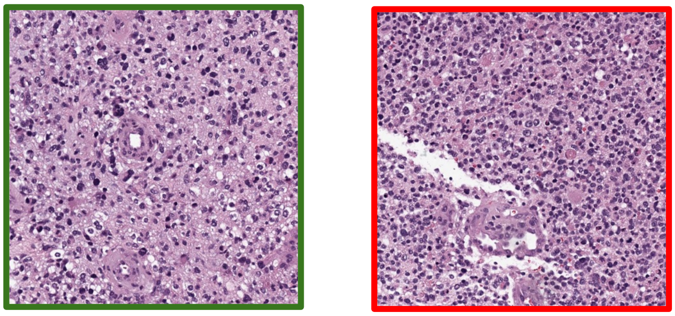

Expert Interpretation: Tumor (GREEN) vs. Tumor (RED) - Moderate Heterogeneity
DLFV r (GREEN vs. RED): 0.75

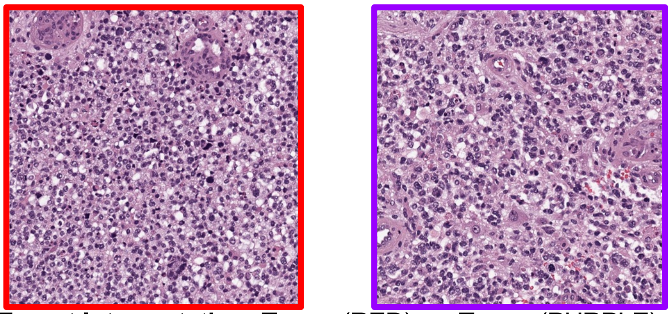

Expert Interpretation: Tumor (RED) vs. Tumor (PURPLE) - Mild/Subjective Heterogeneity
DLFV r (RED vs. PURPLE): 0.80

Fig. S20.

Calibration of DLFV correlation score with human assessment of heterogeneity across HAVOC partitions. (A-C) A representative WSI (panel **A**) with related HAVOC map (panel **B**) and DLFV correlation matrix (panel **C**). Scale: dimensions of image patches shown in panel **B** is 0.066 mm^2. To calibrate DLFV partition correlation scores with human perceivable levels of heterogeneity, histology-trained members on our team examined and scored paired tissue patterns across HAVOC partitions and compare their overall assessments with the DLFV correlation score to generated in panel **C**. **(D)** Demonstrative examples of how regions were scored. The following definitions were generated for consistency: A heterogeneity class of "severe" was defined as "tumor partitions showing obvious pattern differences at low magnifications (5-10x)". A "moderate" heterogeneity class was defined as "tumor partitions showing obvious pattern differences at higher magnifications" (20-40x). A "mild" classification was reserved for "tumor partitions showing only mild and/or subjective pattern differences; even at higher magnification". Tumor vs normal comparisons were also included as a reference and were defined as region pairs clearly comprised of a tumor and normal tissue partition. Dimensions of image patches shown: 0.27 mm^2. Note the multiple unique pair comparisons possible within individual WSIs.

Figure S21

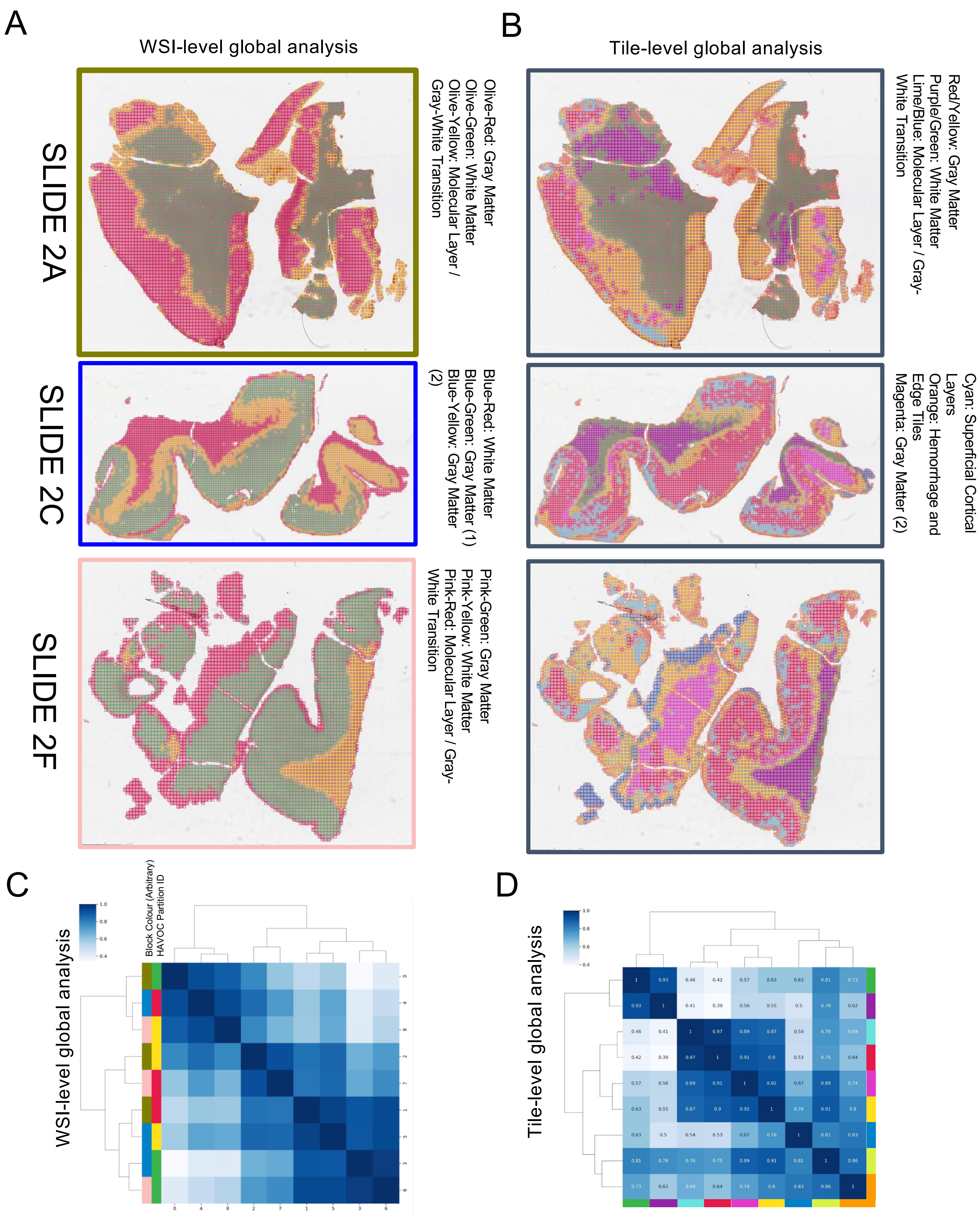
A
WSI-level global analysis
SLIDE 2A
Olive-Red: Gray Matter
Olive-Green: White Matter
Olive-Yellow: Molecular Layer / Gray-White Transition
SLIDE 2C
Blue-Red: White Matter
Blue-Green: Gray Matter (1)
Blue-Yellow: Gray Matter (2)
SLIDE 2F
Pink-Green: Gray Matter
Pink-Yellow: White Matter
Pink-Red: Molecular Layer / Gray-White Transition
B
Tile-level global analysis
Red/Yellow: Gray Matter
Purple/Green: White Matter
Lime/Blue: Molecular Layer / Gray-White Transition
Cyan: Superficial Cortical Layers
Orange: Hemorrhage and Edge Tiles
Magenta: Gray Matter (2)
C
WSI-level global analysis
Block Colour (Arbitrary)
HAVOC Partition ID
D
Tile-level global analysis

Fig. S21.

Comparison of WSI- and tile-level global specimen-level heterogeneity analysis with HAVOC. Demonstrative example of 3 related WSIs from a large epilepsy resection specimen to highlight two different strategies for multi-slide HAVOC analysis. **(A)** In the "WSI-level global analysis", each WSI is partitioned into 3 HAVOC regions for a total of 9 HAVOC regions over the 3 WSIs. Slides in this approach are given arbitrary slide colors (border) to distinguish partitions across WSI with the same HAVOC partition color. **(B)** In the "tile-level global analysis", the totality of tiles across the 3 WSIs are distributed in a single step into 9 HAVOC partitions for comparison. The tile size was set to 512 pixel dimensions in both cases (0.066 mm^2). (**C-D**) Overall, the correlation matrices show, that for both approaches, the major patterns of significant biovariation is in the separation of gray and white matter. Even with this fairly simple example, the relative intermixing of the subregions in the "tile-level global analysis", even when valid, would create critical technical limitations for our benchmarking strategies. While there may be some increased subjectivity in choosing the optimal number of HAVOC partitions in the tile-level global analysis, especially as the number of slides and their complexity increases, both approaches have different strengths and weakness. For future applications of HAVOC, either method could be used based on specific needs and preferences.

Table S1

Patient ID (Block)	Primary vs Secondary	Age	Sex	Location	IDH Status	IDH Method	ATRX (IHC)	p53 (IHC)	MGMT Promoter Methylation (Sequencing)	Diagnosis
I (Block 1B)	Primary	67	Female	Right temporal	WT	IHC	Retained	WT	Positive	Glioblastoma, WHO Grade 4
IIa (Block 3C)<br>IIb (Block 3D)	Primary	49	Male	Right temporal	WT	Sequencing	Retained	WT	N/A	High grade glioma (WHO grade 3)
IIIa (Block 1A)<br>IIIb (Block 1B)	Primary	57	Male	Right parietal/ frontal	WT	IHC	Retained	Mutant	Negative	Glioblastoma, WHO Grade 4
IV (Block 2F)	Primary	52	Male	Right parietal/ frontal	WT	IHC	Retained	WT	Negative	Glioblastoma, WHO Grade 4
Va (Block 2A)<br>Vb (Block 2D)	Primary	55	Female	Right frontal	WT	IHC	Retained	WT	Positive	Glioblastoma, WHO Grade 4
VI (Block 2C)	Primary	56	Male	Left parietal/ occipital	WT	IHC	Retained	WT	N/A	Glioblastoma, WHO Grade 4

Table S1.

Clinical and pathological details of high-grade glioma cohort used for HAVOC-based regional profiling.

Table S2

Patient ID	Primary vs Secondary	Age	Sex	Location	IDH Status	IDH Method	ATRX (IHC)	p53 (IHC)	MGMT Promoter Methylation (Sequencing)	Diagnosis
F1C (Block 2E)	Primary	54	M	Right temporal	WT	Sequencing	Retained	WT	Negative	Anaplastic Astrocytoma, IDH-wildtype, WHO Grade 3
F2A (Block 2A)	Primary	67	Female	Right temporal	WT + BRAFV600 E Subclone	Sequencing	Retained	WT	Negative	Glioblastoma, WHO Grade 4
F2B (Block 2A)	Primary	67	Male	Left frontal	WT	IHC	Retained	N/A	Positive	Glioblastoma, WHO Grade 4
F2H, SF7-II (Block 2B)	Primary	56	Male	Left temporal	WT	IHC	Retained	WT	Negative	Glioblastoma, WHO Grade 4
F3B (Blocks 1A-1L)	Recurrent	51	Male	Left frontal	Mutant	IHC	Retained	WT	N/A	Anaplastic Oligodendroglioma. IDH-mutated, 1p19q codeleted, WHO Grade 3
SF7-I (Block 1A)	Primary	57	Male	Left parietal	WT	IHC	Retained	N/A	Negative	Glioblastoma, WHO Grade 4
SF7-III (Block 2C)	Primary	63	Male	Right frontal	WT	IHC	Retained	Mutant	Positive	Glioblastoma, WHO Grade 4
SF7-IV (Block 2F)	Primary	60	Male	Left frontal	Mutant	IHC	Retained	WT	N/A	Anaplastic Oligodendroglioma. IDH-mutated, 1p19q codeleted, WHO Grade 3
SF7-V (Block 2A)	Primary	68	Male	Right frontal	WT	IHC	Retained	Mutant	Negative	Glioblastoma, WHO Grade 4

Table S2.

Clinical and pathological details of additional representative cases analyzed by HAVOC. For convenience, Patient ID represents figure (e.g. F1) and panel (e.g. C) where this case was introduced. N/A denotes not available.

Table S3

7 Cluster Solution
Cluster 1:

**Expert Annotations**	Observed	Expected
Leading Edge of Tumor	9	1.7143
Moderate Cellularity	2	3.2857
Normal White Matter	1	1.2857
High Cellularity	0	1.7143
Hemorrhage	0	0.7143
Artifacts	0	1.4286
Nodule	0	1.8571

Chi-Square Test
Expert Annotations P-val: 1.5773E-06
Positional Coordinates P-val: 8.7336E-01

Cluster 2:

**Expert Annotations**	Observed	Expected
Leading Edge of Tumor	3	1.5714
Moderate Cellularity	0	3.0119
Normal White Matter	8	1.1786
High Cellularity	0	1.5714
Hemorrhage	0	0.6548
Artifacts	0	1.3095
Nodule	0	1.7024

Chi-Square Test
Expert Annotations P-val: 7.3525E-09
Positional Coordinates P-val: 5.5507E-01

Cluster 3:

**Expert Annotations**	Observed	Expected
Leading Edge of Tumor	0	1.1429
Moderate Cellularity	0	2.1905
Normal White Matter	0	0.8571
High Cellularity	1	1.1429

Hemorrhage	1	0.4762
Artifacts	0	0.9524
Nodule	6	1.2381

Chi-Square Test
Expert Annotations P-val: 5.1090E-04
Positional Coordinates P-val: 9.6992E-01

Cluster 4:

**Expert Annotations**	Observed	Expected
Leading Edge of Tumor	0	1.0000
Moderate Cellularity	7	1.9167
Normal White Matter	0	0.7500
High Cellularity	0	1.0000
Hemorrhage	0	0.4167
Artifacts	0	0.8333
Nodule	0	1.0833

Chi-Square Test
Expert Annotations P-val: 4.9645E-03
Positional Coordinates P-val: 6.7448E-01

Cluster 5:

**Expert Annotations**	Observed	Expected
Leading Edge of Tumor	0	1.7143
Moderate Cellularity	0	3.2857
Normal White Matter	0	1.2857
High Cellularity	9	1.7143
Hemorrhage	0	0.7143
Artifacts	0	1.4286
Nodule	3	1.8571

Chi-Square Test
Expert Annotations P-val: 4.3612E-07
Positional Coordinates P-val: 9.6992E-01

Cluster 6:

**Expert Annotations**	Observed	Expected
Leading Edge of Tumor	0	2.5714
Moderate Cellularity	14	4.9286

Normal White Matter	0	1.9286
High Cellularity	2	2.5714
Hemorrhage	0	1.0714
Artifacts	0	2.1429
Nodule	2	2.7857

Chi-Square Test
Expert Annotations P-val: 3.7828E-04
Positional Coordinates P-val: 9.8555E-01

Cluster 7:

**Expert Annotations**	Observed	Expected
Leading Edge of Tumor	0	2.2857
Moderate Cellularity	0	4.3810
Normal White Matter	0	1.7143
High Cellularity	0	2.2857
Hemorrhage	4	0.9524
Artifacts	10	1.9048
Nodule	2	2.4762

Chi-Square Test
Expert Annotations P-val: 4.8215E-10
Positional Coordinates P-val: 9.3117E-01

3 Cluster Solution
Cluster 1:

**Expert Annotations**	Observed	Expected

Leading Edge of Tumor	12	3.2857
Moderate Cellularity	2	6.2976
Normal White Matter	9	2.4643
High Cellularity	0	3.2857
Hemorrhage	0	1.3690
Artifacts	0	2.7381
Nodule	0	3.5595

Chi-Square Test
Expert Annotations P-val: 6.3265E-10
Positional Coordinates P-val: 7.3263E-01

Cluster 2:

**Expert Annotations**	Observed	Expected
Leading Edge of Tumor	0	6.4286
Moderate Cellularity	21	12.3214
Normal White Matter	0	4.8214
High Cellularity	12	6.4286
Hemorrhage	1	2.6786
Artifacts	0	5.3571
Nodule	11	6.9643

Chi-Square Test
Expert Annotations P-val: 2.6038E-05
Positional Coordinates P-val: 9.7541E-01

Cluster 3:

**Expert Annotations**	Observed	Expected
Leading Edge of Tumor	0	2.2857
Moderate Cellularity	0	4.3810
Normal White Matter	0	1.7143
High Cellularity	0	2.2857
Hemorrhage	4	0.9524
Artifacts	10	1.9048
Nodule	2	2.4762

Chi-Square Test
Expert Annotations P-val: 4.8215E-10

Positional Coordinates P-val: 9.3117E-01

<u>2 Cluster Solution</u>

Cluster 1:

Expert Annotations	Observed	Expected
Moderate Cellularity	0	3.7333
High Cellularity	1	2.1333
Hemorrhage	1	0.1778
Nodule	6	1.9556

Chi-Square Test
Expert Annotations P-val: 8.9419E-04
Positional Coordinates P-val: 9.6096E-01

Cluster 2:

Expert Annotations	Observed	Expected
Moderate Cellularity	21	17.2667
High Cellularity	11	9.8667
Hemorrhage	0	0.8222
Nodule	5	9.0444

Chi-Square Test
Expert Annotations P-val: 3.1203E-01
Positional Coordinates P-val: 9.9997E-01

Table S3.

Contingency tables and p-values of Chi-square testing for histological pattern enrichments in clusters of HAVOC regions across multiple WSIs shown in Figure 3. Results for each cluster and for all three clustering solutions shown in **Fig S10** are provided. Each distribution is compared to the expected frequencies of each histological pattern within a cluster. The p-value of potential deviation from of Slide IDs (WSIs ID) in each cluster are also provided as a reference.
